# Targeting CPS1 attenuates lung cancer metastasis by regulating EMT through an epigenetic mechanism

**DOI:** 10.7150/thno.123679

**Published:** 2026-01-01

**Authors:** Yi Ding, Yuying Tian, Mengjuan Li, Yunyun Fu, Tingting Wang, Xiaoyao Du, Mingming Wang, Lele Dong, Fei Gao, Bei Liu, Yunhan Lu, Chenfei Zhang, Lin Mao, Jinhua Jiang, Lei Li, Lujian Liao, Kun Li

**Affiliations:** 1 Shanghai Key Laboratory of Regulatory Biology, School of Life Sciences, East China Normal University; Shanghai, 200241, China.; 2 Durbrain Medical Laboratory; Hangzhou, 310000, China.; 3 State Key Laboratory of Systems Medicine for Cancer, Shanghai Cancer Institute, Renji Hospital, Shanghai Jiao Tong University School of Medicine, Shanghai, China.; 4 Department of Interventional Oncology, Renji Hospital, Shanghai Jiaotong University School of Medicine, Shanghai, 200001, China.; 5 Department of Musculoskeletal Oncology, Fudan University Shanghai Cancer Center, Shanghai 200032, China.; 6 Jiangong Hospital Clinical Research Center, East China Normal University; Shanghai, 200241, China.

**Keywords:** Lung cancer, metastasis, CPS1, epithelial-to-mesenchymal transition, metabolic reprogramming, fumarate

## Abstract

**Background:** Metastasis is the primary cause of cancer-related mortality, and targeting the drivers of this process is a promising strategy to improve patient outcomes. Recent studies have highlighted a role of Carbamoyl Phosphate Synthetase 1 (CPS1), the urea cycle's rate-limiting enzyme, in tumor development. However, its involvement in tumor spreading and metastasis remains unclear.

**Methods:** Transwell assay, wound healing assay and a range of lung cancer metastasis animal models were employed to investigate the impact of genetic knockdown and pharmacological inhibition of CPS1 on lung cancer metastasis both *in vitro* and *in vivo*. Quantitative proteomic analysis, RNA sequencing, untargeted metabolomics and targeted metabolomics to urea cycle were conducted to elucidate the underlying mechanisms of CPS1 inhibition.

**Results:** CPS1 was overexpressed in a subset of patients with metastatic lung cancer, and this increased expression correlated with decreased patient survival. Genetic knockdown and pharmacological inhibition of CPS1 significantly reduced the tumor burden and metastasis in mice with the spontaneous (*Kras*^G12D/+^; *p53*^-/-^) and induced metastatic lung cancer. Mechanistically, CPS1 overexpression in metastatic cancer cells resulted in excessive fumarate production, an intermediate metabolite in the urea cycle. Fumarate accumulation inhibited TET2 activity and altered *miR200a* gene methylation to drive epithelial-to-mesenchymal transition (EMT), thereby enhancing cell migration and invasion. Notably, CPS1 inhibition reduced fumarate accumulation and enhanced TET2 activity, which epigenetically upregulated PD-L1 expression. This activation contributed to impaired CD8⁺ T cell function and ultimately promoted tumor immune evasion. To overcome immune evasion, we investigated a combination therapy. Combining a CPS1 inhibitor with an anti-PD-1 antibody demonstrated a synergistic and potent effect, significantly inhibiting both lung tumor growth and metastasis.

**Conclusions:** These findings define a crucial role for CPS1 in lung cancer metastasis. Targeting CPS1 may offer a valuable therapeutic intervention strategy against metastatic lung cancer.

## Introduction

Lung cancer is a devastating disease with the highest incidence and mortality rate among all types of cancers 1. Metastasis, the spread of cancer cells from the primary tumor in the lung to other organs, particularly the bone and the brain, is the main reason for high mortality rate, especially in non-small cell lung cancer (NSCLC) 2. Precision medicine has revolutionized treatment strategies for metastatic lung cancer. Small molecule inhibitors targeting common genetic mutations like epidermal growth factor receptor (*EGFR*) and anaplastic lymphoma kinase (*ALK*) have achieved significant success 3, 4. Recent studies have also shown that combining immune checkpoint inhibition with traditional treatments like surgery, chemotherapy, or radiation therapy leads to significantly longer survival 2. Despite these advances, a large percentage of lung cancer patients do not response to these treatments. Therefore, identifying new mechanisms that drive lung cancer metastasis is crucial for developing innovative and more effective treatment approaches.

During metastasis, cancer cells adapt their metabolism to support their growth and survival in new tissue environments 5-7. Carbamoyl phosphate synthase 1 (CPS1), the rate-limiting enzyme in the urea cycle primarily found in the liver, converts ammonia and bicarbonate into carbamoyl phosphate in mitochondria 8. Although its primary role is detoxification of ammonia in the liver 9, CPS1 is also detected in various cancers 10. In some contexts, loss of the tumor suppressor kinase *LKB1* leads to upregulation of CPS1. This can interfere with pyrimidine biogenesis, essential building blocks for DNA, potentially causing DNA damage and promoting cell proliferation 11. Studies have shown a link between *LKB1*-negative lung adenocarcinoma (LADC) patients and CPS1 expression 12, suggesting a potential role for CPS1 in this specific cancer subtype. Further research indicates that mutation in *p53* also led to the dysregulation of CPS1 and the urea cycle, impacting the biosynthesis of polyamines and nucleotides necessary for cancer growth and progression 13. Interestingly, in a subtype of hepatocellular carcinoma (HCC) where CPS1 expression is low, cells exhibit elevated ammonia levels, which subsequently activate pathways like AMPK-FOXM1 axis, enhancing lipid oxidation and ATP production to fuel tumor growth. This low CPS1 expression in HCC is associated with a poor prognosis 14. Despite these findings, the precise role of CPS1 in the metastatic process and whether its expression is altered in metastatic tumors remains an open question.

Fumarate, a metabolite with a multifaceted role in cellular processes, also acts as an important epigenetic regulator. In certain cancers, such as renal cell carcinoma with hereditary leiomyomatosis (HLRCC), loss-of-function mutations in fumarate hydratase (FH) lead to accumulation of fumarate. This excess fumarate inhibits DNA demethylases, causing subsequent DNA hypermethylation of the miR200 family of tumor suppressor genes. These microRNAs normally suppress epithelial-mesenchymal transition (EMT) 15, a process where cancer cells acquire increased mobility and invasiveness. The dysregulation of miR200, driven by fumarate accumulation, results in uncontrolled expression of transcription factors like TWIST, SNAIL and ZEB, which promote EMT 16, 17, and is linked to the highly aggressive phenotype observed in FH-deficient renal cell cancers. Fumarate levels are tightly regulated within cells due to its involvement in key metabolic pathways, including the TCA cycle, amino acid metabolism, and nucleic acid biosynthesis 18, 19. These pathways are crucial for preventing uncontrolled cell growth and tumor formation. However, the effect of increased CPS1 activity on fumarate production and its role in tumor progression is not fully understood.

In this study, we investigated the role of CPS1 in promoting lung cancer metastasis and its underlying mechanism. Our study demonstrated that CPS1 was overexpressed in metastatic lung cancer and correlated with poor prognosis. We showed that CPS1 promoted EMT through fumarate accumulation, which inhibited TET2-mediated DNA demethylation of the miR200a promoter, leading to epigenetic silencing and enhanced cell migration. Furthermore, CPS1 depletion or pharmacological inhibition suppressed metastasis in multiple mouse models and synergized with anti-PD-1 immunotherapy to enhance antitumor immunity. These findings establish CPS1 as a key metabolic driver of lung cancer metastasis and highlight its potential as a therapeutic target.

## Materials and Methods

### Cell lines

The NSCLC cell line A549 (L0) and derived metastatic clones (L2, L6) were obtained from professor Luo Jian (Tongji University School of Medicine, China). The cell lines generated in this research and their parental cell lines (L0, L2, L6) were grown in DMEM with 10% fetal bovine serum (FBS) and 100 mg/mL penicillin/streptomycin. H1975, H441, H460, CMT167 cells were grown in RPMI1640 with 10% FBS and 100 mg/mL penicillin/streptomycin. All cells were maintained at 37 °C with 5% CO_2_ in cell incubator.

### Human lung cancer tissues and paraffin-embedded samples

Frozen tumor specimens for western blot analysis (n = 10, 12, 10 for para-tumor, primary tumor, and metastatic tumor tissues, respectively) and for PDO were obtained from the Department of Musculoskeletal Oncology in Fudan University Shanghai Cancer Center with informed patient consent and the approval from Fudan Institutional Review Boards. Paraffin-embedded primary and metastatic tumors for IHC were also obtained from the Department of Musculoskeletal Oncology in Fudan University Shanghai Cancer Center with informed patient consent and approval from the Hospital Institutional Research Ethics Committee (050432-4-2108*).

### Mouse experiments

Sex-randomized mice were used in the study. BALB/c nude mice, C57BL/6 mice aged 6 weeks were used to establish a lung cancer metastasis model in this study, and they were provided by East China Normal University purchased from Jihui Animal Feeding Co, Ltd (Shanghai, China). All animal protocols were approved by East China Normal University and were performed in accordance with the guidelines of the Ethics Committee of East China Normal University (m20230902). Mice were caged in the groups of five in a laminar air flow cabinet under specific pathogen-free conditions. They were fed with abundant food and water, and kept on a 12 h light/dark cycle. L6 cells expressing shCPS1 or scrambled shRNA were injected into the left ventricle of the mice for the lung cancer metastasis model. Nude mice were injected with cancer cells at an amount of 1×10^5^ cells per mouse. For H3B-120 treatment, mice were i.p. injected with H3B-120 (40 mg/kg or 80 mg/kg) after left ventricle injection for 28 days since day 7. For PD-1 antibody and H3B-120 treatment, mice were i.p. injected PD-1 antibody (50 μg per mouse) every three days and H3B-120 (80 mg/kg) once daily, starting from day 3 after tail vein injection, for a total of 46 days. Live-animal images were acquired with IVIS Spectrum CT (PerkinElmer).

### Proteomics

Samples were lysed in a buffer containing 8 M urea with protease and phosphatase inhibitors (A32959, Thermo) on ice for 30 minutes. The samples were then subjected to non-contact ultrasound sonication for 5 minutes at 4 °C, and then centrifuged (16000 × g, 15 minutes, 4 °C). Protein concentration of the supernatant was quantified using Bradford assay kit (23246, Thermo), then protein (30 μg) was reduced with dithiothreitol (DTT, 10 mM) at 55 °C for 30 minutes. After being cooled to room temperature, the proteins were alkylated with iodoacetamide (IAA, 15 mM) in the dark for 30 minutes. Afterwards, four volumes of 50 mM NH_4_HCO_3_ solution were added. Samples were digested at 37 °C with trypsin (1:100) (V5111, Promega) overnight. Finally, 1% final concentration of formic acid was added to terminate the enzymatic reaction. The samples were then desalted and dried and resuspended in buffer A (2% ACN, 0.1% formic acid) for LC-MS/MS. For MS data analysis, the MaxQuant software (version 1.4.1.2) was used. MS/MS spectra were searched against a UniportKB/Swiss-Prot human database. The precursor mass tolerance was set to 15 ppm, and trypsin was set as the protease.

### RNA sequencing

Total RNA was extracted from cells with Trizol. RNA concentration was measured with Nanodrop2000 (Nanodrop2000, Thermo). RNA integrity was evaluated with Agient2100 (LabChipGX, PerkinElmer). The mRNA was purified using VAHTSTM DNA Clean Beads (N411-03, Vazyme), followed by the preparation of complementary DNA (cDNA) from the isolated mRNA with the VAHTSUniversalV6RNA-seqLibrary Prep Kit for Illumina®, (NR604-02, Vazyme). The prepared RNA-sequencing libraries underwent next-generation sequencing of 120 bp from both ends (paired-end reads) with a llumina NovaSeqX plus platform (Illumina, San Diego). The sequencing data was filtered using SOAPnuke (v1.5.2), after which the clean reads were mapped to the reference genome with HISAT2 (v2.0.4). Bowtie2 (v2.2.5) was employed to map the clean reads against the reference coding gene set, and StringTie (v2.1.2) was utilized to calculate the gene expression levels.

### Metabolomic assay

Each cell sample (50 mg) was suspended in methanol (400 μL) (containing 5 μg/mL 2-chloro-L-phenylalanine as internal standard). The suspension was vortexed for 1 min and homogenized for 3 minutes at 60 Hz twice. Then the solution was centrifuged at 13000 rpm, 4 °C for 10 min. The supernatant was transferred to sample vials for detection. An equal amount of 20 μL from each sample was taken and mixed as the Quality Control (QC) sample. An Agilent 1290 Infinity Ⅱ UHPLC system coupled to an Agilent 6545 UHD and Accurate-Mass Q-TOF/MS was used for LC-MS analysis. For analysis of the raw data, Agilent Masshunter Qualitative Analysis B.08.00 software (Agilent Technologies, USA) was used for peak extraction, integration, identification, and quantification of each metabolite. R language (v4.3.3) was used for subsequent statistical analysis. All multivariate analyses and modeling on the normalized data were carried out using Metaboanalyst 6.0 (http://www.metaboanalyst.ca). Univariate statistical differences of the metabolites between two groups were analyzed using Student's *t*-test.

### Wound healing assay

Cells were plated onto a 12-well dish and cultured in standard conditions until the density reached 100%. The cells were scratched with a pipette tip and replaced with serum-free culture medium. Scratches were recorded at the same location at 0, 24 and 48 h after scratching using a microscope, and the scratched area was measured and quantified using Image J.

### Transwell migration assay

For each experimental condition 1×10^5^ cells were plated onto the chamber of transwell inserts (3422, Corning) containing serum-free culture medium. Complete medium with 10% fetal bovine serum was used as an attractant. After 36 h of migration, the migrated cells were fixed with 4% paraformaldehyde (PFA) and stained with 0.5% crystal violet (C8470, Solarbio), then counted using Image J.

### Colony formation assay

Cells were plated onto a 6-well dish (3×10^3^ cells per well) and allowed to adhere overnight. Then the cells were treated with indicated drugs for 7 days. Medium with or without drug were replaced every two days. The remaining cells were fixed with 4% PFA and then stained with 0.5% crystal violet. The relative number of colonies was counted by normalization to untreated group which was set as 100%.

### Detection of global DNA methylation using HPLC

Cells were lysed in buffer (10 mM NaCl, 10 mM EDTA pH 8.0, 20 mM Tris-HCl pH 8.0, 0.5% SDS with 100 μg/mL proteinase K) (10401ES60, Yeasen) at 55 °C overnight. DNA was extracted with phenol:chloroform:isoamyl alcohol 25:24:1 (BSA03M1, BIOER), precipitated in isopropanol at -20 °C for 15 minutes and washed with 75% ethanol, the concentration measured by Nanodrop-2000, then DNA (10 μg) was digested with nuclease P1 (M0660S, NEB) at 37 °C overnight, and finally dephosphorylated with Antarctic phosphatase (M0289S, NEB) at 37 °C for 4 h. Deoxycytosine (dC) and 5-methyl-deoxycytosine (5mdC) were detected with 280 nm UV by an HPLC system (Agilent, 1260 Infinity Ⅱ) with a BEH C18 column (Waters XBridge, 5 μm, 250 mm × 4.6 mm, 186003625). Separation was achieved at a flow rate of 0.5 mL/min according to the following linear gradient: 0%-70% buffer B (100% MeOH) for 25 min, 70%-0% buffer B for 20 min. Buffer A was composed of KH_2_PO_4_ (10 mM), pH3.6. Nucleotides were analyzed by OpenLAB CDS ChemStation based on peaks areas.

### Methyl specific PCR (MSP) assay

Purified DNA (1 μg) were converted by bisulphate using the DNA Methylation Bisulfite Kit (EM101, Vazyme) following the manufacturer's instruction. PCR of converted DNA was performed using 2 × EpiArt HS Taq Master Mix (EM201, Vazyme) following manufacturer's protocol. The PCR product was separated by agarose gel electrophoresis and images were obtained using Gel Image System. Methylation-specific primers were designed using MethPrimer and are listed in the Table S7.

### TET2 enzymatic activity

TET2 activity in cells (1×10^6^ cells) was measured using the TET2 chemiluminescent assay kit (50652, BPS Bioscience), following the manufacturer's instructions. The demethylation level of hydroxymethylated substrates was measured using a chemiluminescence reader (Synergy NEO2, BioTek) 20.

### Western blot

Cultured cells were collected by trypsin and then lysed with RIPA (P0013C, Beyotime) lysis buffer (20 mM Tris-HCl pH7.6, 150 mM NaCl, 1% NP-40 detergent, 1% sodium deoxycholate, 0.1% SDS with phosphatase and protease inhibitors), and frozen tissue were homogenized in RIPA lysis buffer for 15 min, followed by centrifugation at 16000 × g for 15 min. The protein concentration was measured with a BCA Protein Assay Kit. Proteins (10 μg) were loaded to determine different proteins and separated on 8% or 10% SDS-PAGE gel. Proteins were transferred from gel to NC membranes. Membranes were then incubated in blocking buffer (5% Bovine Serum Albumin in TBST) for 1 h at room temperature. Primary antibodies were incubated overnight at 4 °C. Secondary antibodies were incubated for1 h at room temperature. Images were captured by Odyssey software (Li-Cor). Antibodies used were β-actin (# 18703-1-AP, 1:1,000; Proteintech), CPS1 (#18703-1-AP, 1:1,000; Proteintech), E-cadherin (#20874-1-AP, 1:10,000; Proteintech), N-cadherin (#22018-1-AP, 1:5,000, Proteintech), TET2 (#21207-1-AP, 1:1,000, Proteintech), Twist (#25465-1-AP, 1:1,000, Proteintech), DNMT1 (#24206-1-AP, 1:1,000, Proteintech), DNMT3A (#20954-1-AP, 1:1,000, Proteintech), DNMT3B (#26971-1-AP, 1:1,000, Proteintech), PD-L1 (#28076-1-AP, 1:1,000, Proteintech), OGDH (#A22163, 1:1,000, ABclonal), GAPDH (#AC002, 1:5,000, ABclonal); ASL (#A00742-1, 1:1,000, Boster), ZEB1 (#3396T, 1:1,000, Cell signaling technology), Snail1 (#3879T, 1:1,000, Cell signaling technology).

### Histology and immunofluorescence

Femurs and spines from nude mice were fixed in 4% PFA for 48 h, then flushed under flowing water for 4-5 h followed by decalcification in 0.5 M EDTA (15575020, Thermo) for 2-3 weeks at 4 °C. Tissues were cut into 6 mm sections followed by H&E staining. Sections were dewaxed and rehydrated, stained with hematoxylin and eosin according to the standard protocol. For immunofluorescent analysis of cultured cells, cells were fixed with 4% PFA for 1 h and treated with 0.4% Triton X-100 (P0096-100, Beyotime) for 15 minutes. The proteins in the permeabilized cells were neutralized with 100 mM Tris-HCl (pH 8.5) for 10 min, and then incubated with blocking buffer (3% bovine serum albumin in PBS containing 0.1% TritonX-100) for 1 h. Cells were incubated overnight at 4 °C with primary antibodies against 5hmC. Alexa Flour 594 were added and incubated for 1 h at room temperature. The nuclei were stained with DAPI (P0131, Beyotime) for 5 min. The coverslips with cells were inverted onto a glass slide filled with antifade mounting medium. Images were acquired by two-photon laser confocal microscope (Leica SP8, Germany).

### Paraffin-embedded specimens Immunohistochemistry (IHC) assay

Paraffin embedded sections were deparaffinized and rehydrated. Antigen retrieval was performed by EDTA at 100 °C in a water bath for 30 minutes. The sections were cooled to room temperature, washed three times with PBS, then incubated with 3% H_2_O_2_ for 10 minutes for quenching endogenous peroxidase activity. Next, antigens were blocked by incubating in PBS containing 5% BSA for 30 minutes. Then, sections were incubated with respective antibodies at 4 °C overnight. DAB substrate kit was applied for color development followed by counterstaining with hematoxylin. For immunofluorescent analysis, secondary antibodies conjugated with fluorophores were added, and the sections were incubated for 1 h at room temperature in darkness. Finally, the sections were microphotographed with an Olympus microscope system and analyzed with Image J software.

### Patient-derived organoid (PDO) assay

Tumor tissues derived from surgical resections were cut into small pieces and enzymatically digested using type I collagenase (17100017, Gibco). Cells were further disassociated with shaking for 30 min at 37 °C until the liquid was cloudy, ground and filtered with 100 μm filtering set. The resulting cells were centrifuged with 1500 rpm for 5 min and the supernatant was discarded. Red blood cells were lysed with lysis buffer (00-4333-58, Thermo Fisher) for 2 minutes on ice, then centrifuged with 1500 rpm for 5 minutes. The resulting cells were washed with PBS (10% FBS and 2% penicillin/streptomycin), and then cell number was counted before embedding in Matrigel (354248, Corning). After solidification for 15 min at 37 °C, cells were overlaid with human lung cancer organoid medium. *In vitro* organoid killing assay was performed when the size of organoids reached 30-100 μm. Calcein-AM/PI (92210, Merck) was used to measure the survival rate of organoids. Fluorescence images were taken and analyzed with Image J.

### Flow cytometry assay

Single-cell suspensions from CMT167 tumors were minced and incubated in a digestion buffer containing 10 U/mL Collagenase type I (17100017, Gibco), 100 U/mL Collagenase type IV (17104019, Gibco), 1 mg/mL DNaseI (10104159001, Sigma), HBSS (PB180324, Pricella) at 37 °C for 45 min with gentle agitation. Post-digestion, the cell suspension was filtered through a 70 μm filter screen to remove debris and treated with red blood cell lysis buffer (C3702, Beyotime). For surface staining, we mixed the appropriate antibodies with the cells at room temperature for 30 min and washed with FACS buffer (PBS supplemented with 2% FBS) for 30 minutes at room temperature. For intracellular staining, cells were fixed with 4% PFA and permeabilized using Perm Wash Buffer (421002, Biolegend) according to the manufacturer's instructions. Flow cytometric analysis was performed on a flow cytometry (BD Fortessa), and data were analyzed using FlowJo software (BD), with compensation and gating strategies applied to exclude doublets and dead cells. The antibodies used were as follows: CD3ε (100306, BioLegend), CD80 (104733, BioLegend), CD45 (103132, BioLegend) IFN-γ (505808, BioLegend), CD4 (740122, BD Biosciences), CD25 (552880, BD Biosciences), Foxp3 (562996, BD Biosciences), F4/80 (123115, BioLegend), CD11b (101205, BioLegend), Fixable Viability stain 780 (565388, BD Biosciences).

### Micro-CT analysis

The spine and femurs from nude mice were separated and fixed in 4% PFA for two days, PFA was changed every 48 h. The bones were scanned by high-resolution X-ray microtomography *in vitro* (SkyScan 1272, Bruker; Belgium) with 60 kV and 166 µA and using a detection pixel size of 9 µm. NRecon software (Bruker) and CTAnalyser software (Bruker) were used to reconstruct and analyze the scanned images respectively. The scanner used Dataviewer (Bruker) and CTVox (Bruker) to make 2D and 3D visual pictures respectively. The trabecular bone of the vertebra and the femur were analyzed, the data included bone volume fraction (BV/TV), bone mineral density (BMD), trabecular thickness (Tb. Th), trabecular number (Tb. N), trabecular separation (Tb. Sp) The LoxP-stop-loxP*Kras^G12D/+^*/FVB/129 *Trp53^-/-^* mice and LoxP-stop-loxP*Kras*^G12D^/FVB/129 *Trp53^-/-^*/*Cps1^-/-^* mice were scanned by inviscan IRIS PET/CT with the Tube Voltage of 80 kV and Vox size of 60 µm. Hiscan Analyzer software (Version 3.0, Suzhou Hiscan Information Technology Co., Ltd) was used to reconstruct and analyze the scanned images respectively.

### LoxP-stop-loxP *Kras^G12D/+^*; *Trp53^-/-^*; *Cps1*^*-/-*^ mouse models

LoxP-stop-loxP*Kras^G12D/+^*/FVB/129 *Trp53^-/-^* mice (KP mice) were crossed with *CPS1^-/-^* mice, yielding LoxP-stop-loxP*Kras*^G12D^/FVB/129 *Trp53^-/-^*; *Cps1^-/-^* (KP *Cps1^-/-^*) mice. Six-week-old KP *CPS1^-/-^*mice were inoculated with 1 × 10^6^ PFU adenoviral Cre (adeno-Cre) by intranasal inhalation to activate oncogenic *p53^-/-^*, *Kras^ G12D/+^* and *CPS1^-/-^* in the lungs.

### Pharmacokinetics and toxicity studies of H3B-120 in mice

Six-week-old ICR Outbred Mice (ICR) were purchased from Beijing Vital River Laboratory Animal Technology and were used in pharmacokinetics study. H3B -120 was injected via tail - vein injection with 2 mg/kg and intraperitoneal with 20 mg/kg, respectively. After injecting the inhibitor, 0.05 mL of blood was collected from the orbital area at 5 minutes, 15 minutes, 30 minutes, 1 h, 2 h, 4 h, 6 h, 8 h and 24 h; The concentration of H3B-120 in mouse plasma samples was determined by LC-MS/MS, and the pharmacokinetic parameters were calculated using WinNolin software. Absolute bioavailability calculation formula: F (%) = (Doseiv × AUCoral (0-∞)) / (Doseoral × AUCiv (0-∞)) × 100%.

H3B-120 was intraperitoneally injected at 3-days intervals for a total of 14 days to ICR mice. Clinical signs, body weight, food consumption was monitored throughout the study. Blood collection from the eye socket was performed on the day after the last administration. AST and ALT were detected in the blood to evaluate the toxicity of the inhibitor.

### Statistical analysis

All statistical calculation was performed using Graphpad Prism 10.5. One-way ANOVA followed by Dunnett t-tests or two-tail paired Student's *t*-tests were used to compare the means among groups *in vitro*. Two-way ANOVA was used to compare experiment groups *in vivo*. For survival analysis Kaplan-Meier estimator with log-rank test was applied to test the differences between two groups. Cox proportional hazard analysis was followed to generate the hazard ratios. Significance was set at *P < 0.05, **P < 0.01 and ***P < 0.001. All the data are available.

## Results

### CPS1 is overexpressed in metastatic lung cancer and is correlated with poor prognosis

**Figure 1:**
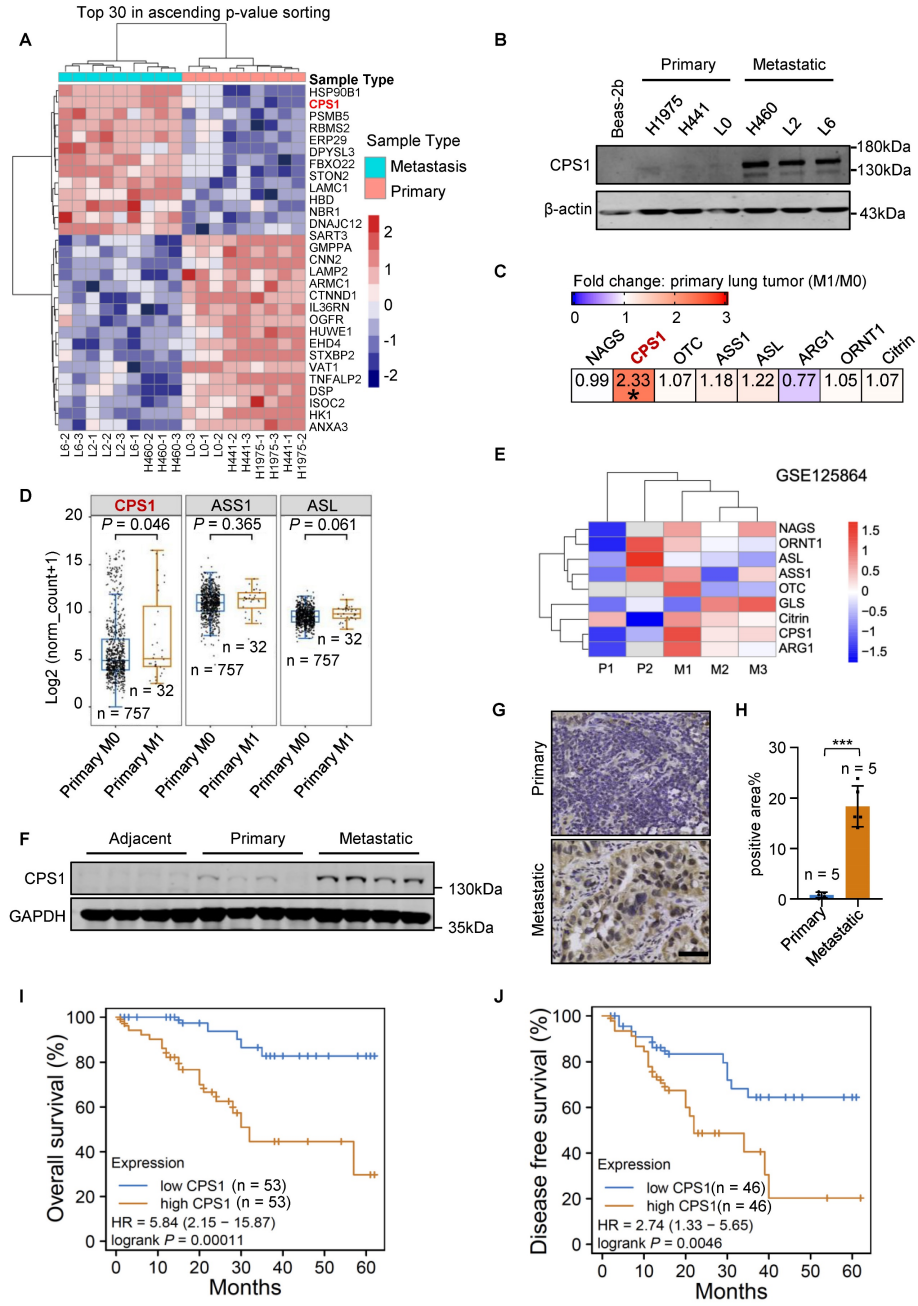
**CPS1 is overexpressed in metastatic lung cancer and correlates with poor prognosis.** (**A**) Heatmap of significantly changed proteins in primary H1975, H441, A549-L0 (L0) and metastatic lung cancer cell lines H460, A549-L2 (L2), A549-L6 (L6). Log2-transformed protein intensities were scaled and clustered using hierarchical clustering. (**B**) Western blot analysis of CPS1 expression in primary and metastatic lung cancer cells. (**C**) mRNA expression of urea cycle enzymes in primary tumors comparing patients with or without metastasis. *P = 0.046. (**D**) Boxplot of mRNA expression of three urea cycle enzymes in primary tumors comparing patients with or without metastasis. (**E**) Heatmap showing mRNA expression of urea cycle enzymes comparing primary and metastatic tumors. (**F**) Western blot analysis of tissue samples from lung cancer patients, including para-tumor tissue (n = 10), primary tumor tissue (n = 12) and tumor tissue from metastatic site in the bone (n = 10). (**G**) Immunohistochemistry of CPS1 in primary tumor and bone metastatic lesions from lung cancer patients. (**H**) CPS1 staining scores are shown. Each value represents mean ± SEM (n = 5), ***P < 0.001. *P* value was calculated by unpaired two-tailed Student's *t*-test. (**I** and** J**) Survival analysis comparing patient survival between CPS1 high versus low expression using 50% as the expression cutoff. Kaplan-Meier univariate log-rank test was applied to estimate the survival. HR was calculated using Cox proportional hazard model. Data were from Gillette MA et al. 2020 (*21*).

The invasive phenotype of the metastatic lung cancer cells (H460, L2, L6) was confirmed relative to primary (H1975, H441, L0) cells using transwell and wound healing assays (Figure S1A-D). Quantitative proteomics showed that CPS1 expression was significantly increased in metastatic cells (Figure 1A, Figure S1E and Table S1), which was consistent with our previous research 21. Western blot analysis confirmed this observation by showing substantial CPS1 overexpression in all metastatic cells compared to primary cells, with negligible expression in the normal lung epithelial cell line Beas-2b (Figure 1B). To further support this finding, transcriptomic data from The Cancer Genome Atlas (TCGA), including lung adenocarcinoma (LUAD) and squamous cell carcinoma (LUSC), was analyzed 4. Patients were categorized as metastatic (M1) or non-metastatic (M0), and significantly increased* CPS1* mRNA was observed in primary cancer tissues of M1 patients (Figure 1C). The other urea cycle enzymes, *ASS1* and *ASL*, showed a minor, non-significant increase (Figure 1C), and the mRNA expression of these three genes is presented in Figure 1D. The dataset GSE125864 from Gene Expression Omnibus (GEO) database also confirmed elevated CPS1 mRNA expression in metastatic compared to primary lung tumor tissues (Figure 1E). Furthermore, Western blot analysis of lung cancer patient samples revealed a significant increase in CPS1 within metastatic tumor tissues (Figure 1F and Figure S1F). Immunohistochemistry studies showed a clear increase in CPS1 expression in metastatic lesions from lung cancer patients (Figure 1G-H).

To explore the clinical ramifications of CPS1 overexpression, Kaplan-Meier survival analysis was performed using protein expression data from the Clinic Proteomic Tumor Analysis Consortium (CPTAC) 22. High CPS1 expression significantly correlated with poor patient overall survival and disease-free survival in lung cancer patient (Figure 1I-J), with average hazard ratios of 5.84 and 2.74, respectively. Furthermore, analysis of TCGA lung cancer data revealed a correlation between high mRNA levels of CPS1 and reduced overall survival, albeit with less dramatic hazard ratios (1.49 for LUAD and 1.34 for LUAD + LUSC) (Figure S1G-H). These findings suggest that CPS1 is overexpressed in some metastatic lung tumors and its overexpression is linked to a poor prognosis.

### Reducing CPS1 expression inhibits lung cancer cell migration and spreading

**Figure 2:**
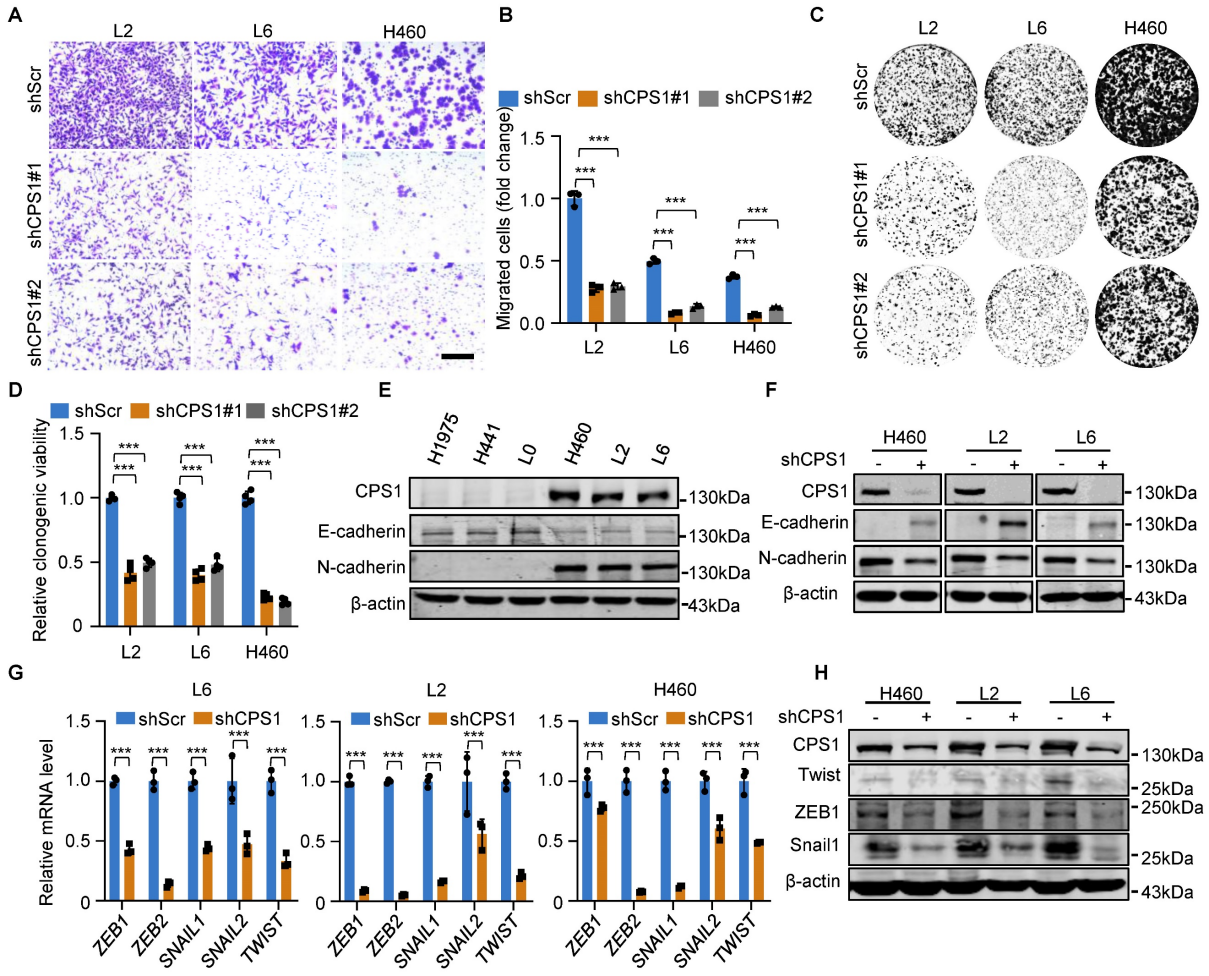
** Reducing CPS1 expression inhibits cancer cell migration and EMT.** (**A**) Representative images of transwell assay showing cell migration after knocking down CPS1. Images were taken 36 h after seeding. Scale bar: 400 μm. (**B**) Statistical analysis of the number of migrated cells in (A). (**C**) Colony formation of metastatic cells after knocking down CPS1 expression. (**D**) Statistical analysis of the colony formation results in (C). (**E**) Western blot analysis of CPS1 expression and EMT markers in primary and metastatic cell lines. (**F**) Western blot analysis of EMT markers after knocking down CPS1. (**G**) Relative mRNA expression of EMT transcription factors after knocking down CPS1. (**H**) Western blot analysis of EMT transcription factors after knocking down CPS1. All the data were analyzed using unpaired Student's *t*-test. Error bars show mean ± SEM. ***P < 0.001.

To metastasize, primary tumor cells must acquire migratory capabilities. Therefore, we investigated the role of CPS1 in promoting cell migration. In contrast to increased migration in metastatic lung cancer cells as shown in Figure S1A-D, knockdown of CPS1 significantly reduced migration in all metastatic cell lines (Figure 2A-B and Figure S2A-B). Colony formation assay also revealed that decreased CPS1 expression significantly reduced colony formation within 7 days (Figure 2C-D), suggesting that CPS1 impacts both the migration and proliferation of metastatic cells.

Given that epithelial-mesenchymal transition (EMT) drives cancer cell migration and spreading, we investigated the role of CPS1 in this process. Metastatic cells exhibited characteristic EMT markers, including significantly reduced E-cadherin expression and dramatically increased N-cadherin expression (Figure 2E). Notably, reducing CPS1 expression in metastatic cells reversed this phenotype, restoring E-cadherin levels and decreased N-cadherin levels (Figure 2F). Furthermore, knocking down CPS1 levels led to the downregulation of key EMT transcription factors, including ZEB, SNAIL, and TWIST (Figure 2G), which was confirmed by Western blot analysis (Figure 2H). These results indicate a strong link between CPS1 and EMT.

### The regulation of EMT by CPS1 involves epigenetic modification of miR200a

**Figure 3:**
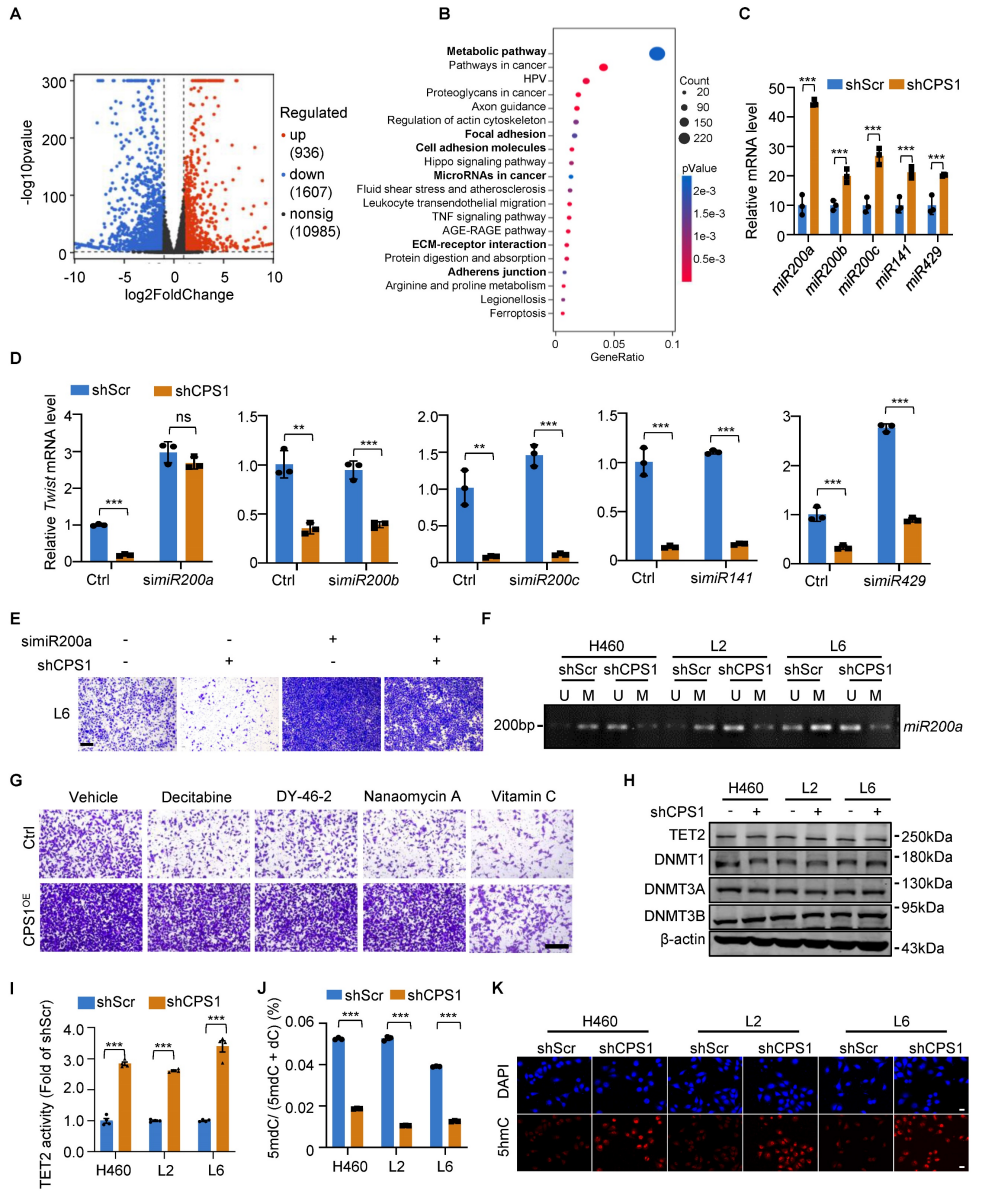
** CPS1 promotes EMT by TET2-induced miR200a methylation.** (**A**) Volcano plot showing the differential mRNA expression levels identified via RNA-sequencing analysis in L6shScr and L6shCPS1 cells. (*P* < 0.05, FC > 1.5 or < 0.67, n = 3) (**B**) Pathway enrichment analysis showing the top 20 significantly differential pathways. (*P*< 0.05, FC > 1.5 or < 0.67, n = 3). (**C**) Relative expression levels of *miR200* family members in L6 cells after CPS1 knockdown. (**D**) Relative mRNA expression levels of *Twist* following sequential knockdown of miR200 family members and CPS1 in L6 cells (**E**) Representative images of transwell assay showing cell migration after knocking down miR200a and CPS1. Images were taken 36 h after seeding. Scale bar: 400 μm. (**F**) Methylation-specific PCR of *miR200a* after CPS1 knockdown. U: un-methylated CpG island; M: methylated CpG island. (**G**) Representative images of transwell assay showing cell migration after treatment with methyltransferases inhibitors and TET2 activators in L0 and L0CPS1^OE^ cells. Decitabine (30 nM): DNMT1 inhibitor; DY-46-2 (1.5 μM): DNMT3A inhibitor; Nanaomycin A (35 nM): DNMT3B inhibitor; Vitamin C (VC) (250 μM): TET2 activator. Images were taken 36 h after seeding. Scale bar: 200 μm. (**H**) Western blot analysis of TET2 and methyltransferases expression in metastatic cells after CPS1 knockdown. (**I**) Enzymatic activity of TET2 after CPS1 knockdown. n = 3. (**J**) Quantification of 5-methyl-deoxycytosine (5mdC) and deoxycytosine (dC) after CPS1 knockdown in metastatic lung cancer cells, n = 3. (**K**) Immunofluorescent imaging of 5hmC (red) and DAPI (blue) staining after knocking down CPS1 in metastatic cancer cells. All data were analyzed using unpaired Student's *t*-test. Error bars show mean ± SEM. **P < 0.01***P < 0.001.

To understand the mechanism of CPS1-mediated lung cancer cell migration, we used RNA-sequencing to compare the transcriptome between L6 cells expressing shRNA targeting CPS1 (shCPS1) and control (shScr). The analysis identified 936 upregulated and 1607 downregulated genes (Figure 3A).

Pathway analysis of the differentially expressed genes revealed that microRNAs in cancer and tumor metastasis-related pathways, including focal adhesion, cell adhesion molecules, ECM-receptor interaction, and adherens junction are among the top 20 changed cellular pathways (Figure 3B and Table S2). Since EMT transcription factors (ZEB, SNAIL, TWIST) are tightly controlled by a family of tumor-suppressor microRNAs particularly the miR200 family^16^, we examined how CPS1 influence the expression level of these microRNAs. After knocking down CPS1 in metastatic cells, while expression of microRNAs potentially involved in EMT including *miR424*^23^, *miR590*^24^, *miR675*^25^, *miR451*^26^, *miR611*^27^, and *miR23a*^28^ showed non-significant or subtle changes (Figure S3A), expression of five miR200 family members increased significantly, with a 5-fold increase in *miR200a* (Figure 3C and Figure S3B). To further determine which miR200 family member is strongly regulated by CPS1, each *miR200* was knocked down in L6 cells stably expressing shCPS1 (Figure S3D). Knocking down CPS1 resulted in a reduction in *Twist* gene expression, likely due to an increase in miRNAs (Figure 3D). In cells with both KD-CPS1 and siRNA against one of the *miR200* member (Figure S3D), only siRNA against miR200a restored *Twist* mRNA to the control level (Figure 3D), indicating that miR200a is the major player regulating Twist expression. Double knockdown of CPS1 and miR200a also showed restoration of *ZEB1* and *Snail1* expression (Figure S3C). While knocking down CPS1 reduced the migration of L6 cells, double knockdown of CPS1 and miR200a restored the migration of L6 cells (Figure 3E and Figure S3E). Importantly, expressing a miR200a mimic (Figure S3F) reduced the migration of L6 cells (Figure S3G-H), increased E-cadherin and reduced N-cadherin (Figure S3I), precisely reversed the EMT phenotype. Thus, these data define a linear CPS1-miR200a signaling pathway that regulates EMT in lung cancer.

To further explore the mechanism behind CPS1's regulation of miR200a expression in metastatic cells, we investigated DNA methylation, a key epigenetic control mechanism for expression of EMT transcripttion factors. Consistent with m*iR200a* downregulation in metastatic lung cancer (Figure S4A), analysis of the *miR200a* promoter revealed significantly higher levels of DNA methylation in metastatic lung cancer cells compared to primary cells (Figure S4B). Knocking down CPS1 effectively reduced DNA methylation of *miR200a* in these metastatic cells (Figure 3F), suggesting that CPS1 induced DNA methylation of *miR200a* to promote the migration of lung cancer cells.

We next investigated the molecular mechanism by which CPS1 regulates *miR200a* DNA methylation to promote cell migration. DNA methylation is primarily regulated by DNA methyltransferases and demethylases, including DNMTs (DNA methyltransferases) and TETs. While transwell assay showed that overexpression of CPS1 in primary lung cancer cells (L0) (Figure S4C) led to increased cell migration (Figure 3G and Figure S4D), vitamin C (VC), known to activate DNA demethylases like TET2 29, dramatically rescued this effect (Figure 3G and Figure S4D). In contrast, treatment with decitabine, DY-46-2, or nanaomycin A, known inhibitors of DNMT1, DNMT3A, and DNMT3B, respectively, did not reverse this enhanced migratory phenotype (Figure 3G and Figure S4D). The expression of TET2, the primary enzyme responsible for converting methylcytosine (mC) to 5-hydroxymethyl-deoxycytosine (5hmC) 30, showed no significant difference between primary and metastatic lung cancer cells (Figure S4E). Meanwhile, CPS1 knockdown did not affect the expression of DNA demethylase TET2, nor the DNMTs (DNMT1, DNMT3A and DNMT3B) (Figure 3H). These findings suggest that CPS1 might influence DNA methylation by altering the enzymatic activity. Indeed, we found that the TET2 activity is significantly higher in primary cells (Figure S4F). High-performance liquid chromatography (HPLC) quantification showed that the ratio of 5mdC to deoxycytosine (dC) was above 4.0% in metastatic cells, significantly higher than the ratio observed in primary cancer cells (below 2.5%) (Figure S4G). Knocking down CPS1 enhanced the enzymatic activity of TET2 (Figure 3I), simultaneously reduced 5mdC/dC ratio in metastatic cells (Figure 3J). In addition, immunofluorescent labeling revealed a notable decrease in 5hmC in metastatic cells (Figure S4H), and the fluorescence intensity of 5hmC was significantly increased by CPS1 depletion (Figure 3K). Collectively, these data establish a cascade wherein CPS1 inhibits TET2 activity, leading to hypermethylation of the miR200a and subsequent activation of the EMT program.

### CPS1 drives lung cancer metastasis by accumulating fumarate to influence TET2 activity and miR200a-EMT axis

In metastatic cells, elevated levels of fumarate may interfere with α-ketoglutarate (α-KG)-dependent dioxygenases, including TET demethylases, thereby reducing its DNA demethylation activity and leading to DNA hypermethylation in downstream genes 17. Our RNA-sequencing analysis also revealed that metabolic pathway was the most significantly enriched pathway following CPS1 knockdown (Figure 3B).

**Figure 4:**
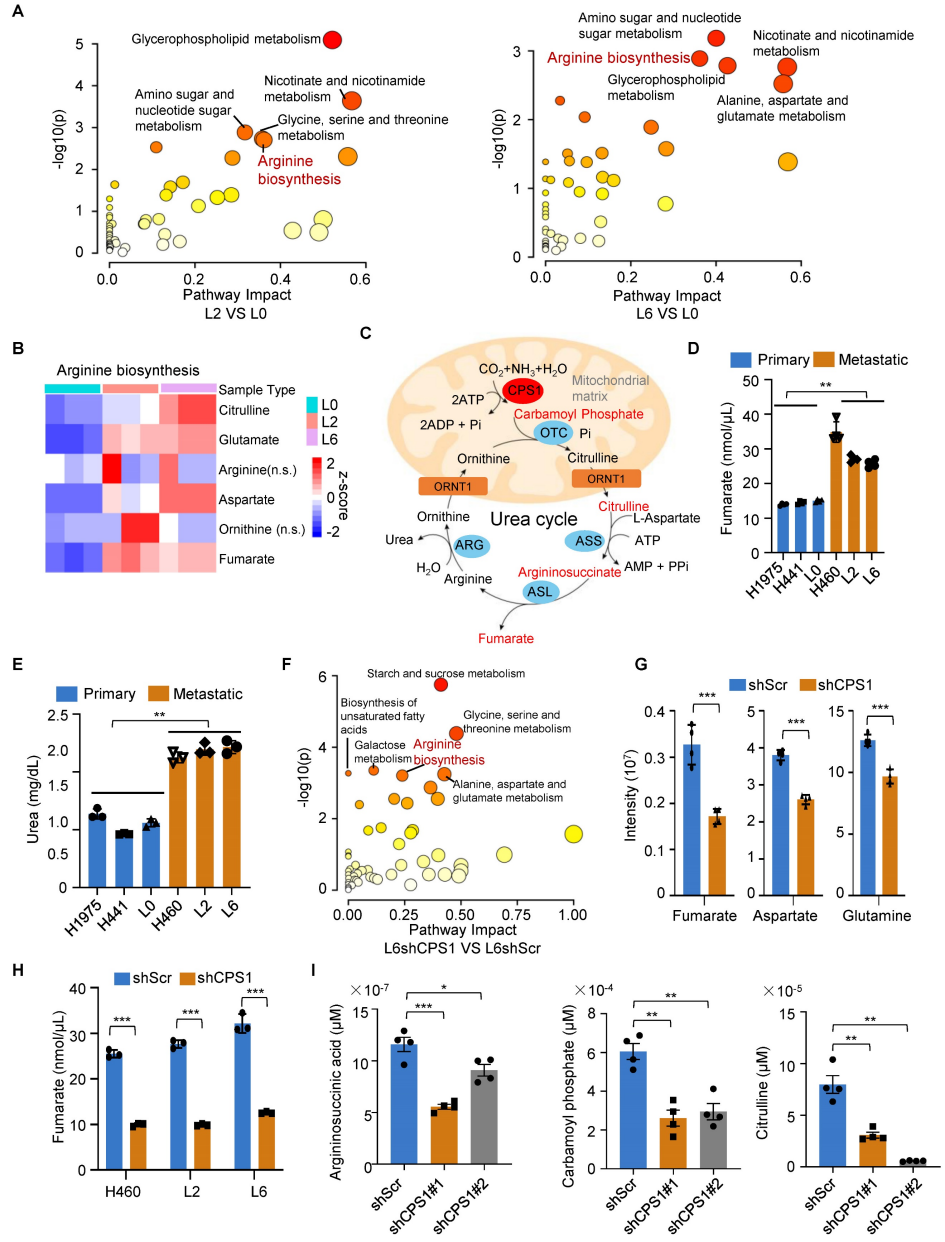
** The level of fumarate is regulated by CPS1 through urea cycle.** (**A**) KEGG pathway analysis of metabolites in primary (L0) and metastatic (L2 and L6) lung cancer cells. Bubbles represent term enrichment. Red: high enrichment, yellow: low enrichment. The sizes of the dots represent the impact of each pathway (KEGG category). *P* < 0.05, VIP score > 1, n = 3 for per group. (**B**) Heatmap illustrating the ratios of metabolites enriched in arginine biosynthesis in KEGG pathway. *P* < 0.05, n = 3 for per group. (**C**) A schematic diagram of the urea cycle metabolic pathway. (**D**) The level of fumarate in primary and metastatic cell lines. n = 4. (**E**) The level of urea in primary and metastatic cell lines. n = 3. (**F**) KEGG pathway analysis of metabolites in L6 cells after knockdown CPS1 expression. The bubble plot showing all metabolic pathways with differential expression. n = 3 for per groups. (**G**) The level of arginine biosynthesis metabolites in L6 cells after knockdown CPS1, n = 4 for per group. (**H**) The level of fumarate after knocking down CPS1 in metastatic lung cancer cells. n = 3. (**I**) Quantification of related metabolites in arginine biosynthesis metabolites in L6 cells after knockdown CPS1. n = 4. All the data were analyzed using unpaired Student's *t*-test. Error bars show mean ± SEM. *P < 0.05, **P < 0.01, ***P < 0.001.

To investigate how CPS1 influences TET2-mediated demethylation in metastatic cells, we performed metabolomic analysis on primary (L0) and metastatic (L2 and L6) lung cancer cells. Quantitative analysis using liquid chromatography-tandem mass spectrometry (LC-MS/MS) revealed significant metabolic alterations between the cell lines, particularly in the arginine biosynthesis pathway. Comparing either L2 to L0 cells or L6 to L0 cells, this pathway was among the top 5 most significantly altered pathways (Figure 4A). Focusing on the metabolites in this pathway, we found consistent increase in fumarate, citrulline, aspartate and glutamate in both metastatic L2 and L6 cells (Figure 4B and Table S3).

Since CPS1 is the rate-limiting enzyme that catalyzes the initial step of the urea cycle, converting ammonia and bicarbonate into carbamoyl phosphate (Figure 4C), we also quantified fumarate (an intermediate metabolite) and urea (the end product). Both metabolites showed a nearly two-fold increase in the metastatic lung cancer cells (Figure 4D-E). Notably, LC-MS/MS revealed that the arginine biosynthesis pathway was downregulated in CPS1-deficient cells (Figure 4F), with a marked reduction in the production of fumarate, aspartate, and glutamine (Figure 4G and Table S4). Reducing CPS1 expression significantly lowered fumarate levels to 30% of the basal level in metastatic cells (H460, L2 and L6) (Figure 4H). Consistent with CPS1's pivotal role in urea cycle, reducing CPS1 expression also significantly lowered the levels of other urea cycle metabolites including argininosuccinic acid, carbamoyl phosphate, and citrulline (Figure 4I and Table S5). Since fumarate is an intermediate metabolic in both the urea cycle and TCA cycle 31, we evaluate the impact on fumarate production by inhibiting the key enzymes in either TCA or urea cycle. Similar reductions of fumarate to 30% of the basal level were observed when argininosuccinate lyase (ASL), another urea cycle enzyme, was knocked down (Figure S5A-B). In comparison, reducing the expression of 2-oxoglutarate dehydrogenase (OGDH), a TCA enzyme responsible for converting α-KG to succinyl-CoA, resulted in a less pronounced reduction of fumarate (to 50-60% of the basal level) (Figure S5C-D). Importantly, CPS1 knockdown did not affect OGDH levels (Figure S5E), indicating that CPS1-mediated changes in fumarate levels are not likely due to altered expression of TCA cycle enzymes. These findings suggest that CPS1 activity plays a more important role in determining fumarate levels in metastatic cells compared to TCA cycle enzymes. Further investigation into TCA intermediate metabolites revealed that only fumarate was substantially increased in metastatic lung cancer cells (Figure S5F-G), potentially due to contributions from other metabolic pathways. Other metabolites, such as pyruvate and phosphoenolpyruvic acid, did not show significant increase in metastatic lung cancer cells (Figure S5F-G). This suggests that CPS1-mediated urea cycle reprogramming likely serves as the primary driver of fumarate accumulation during lung cancer metastasis.

**Figure 5:**
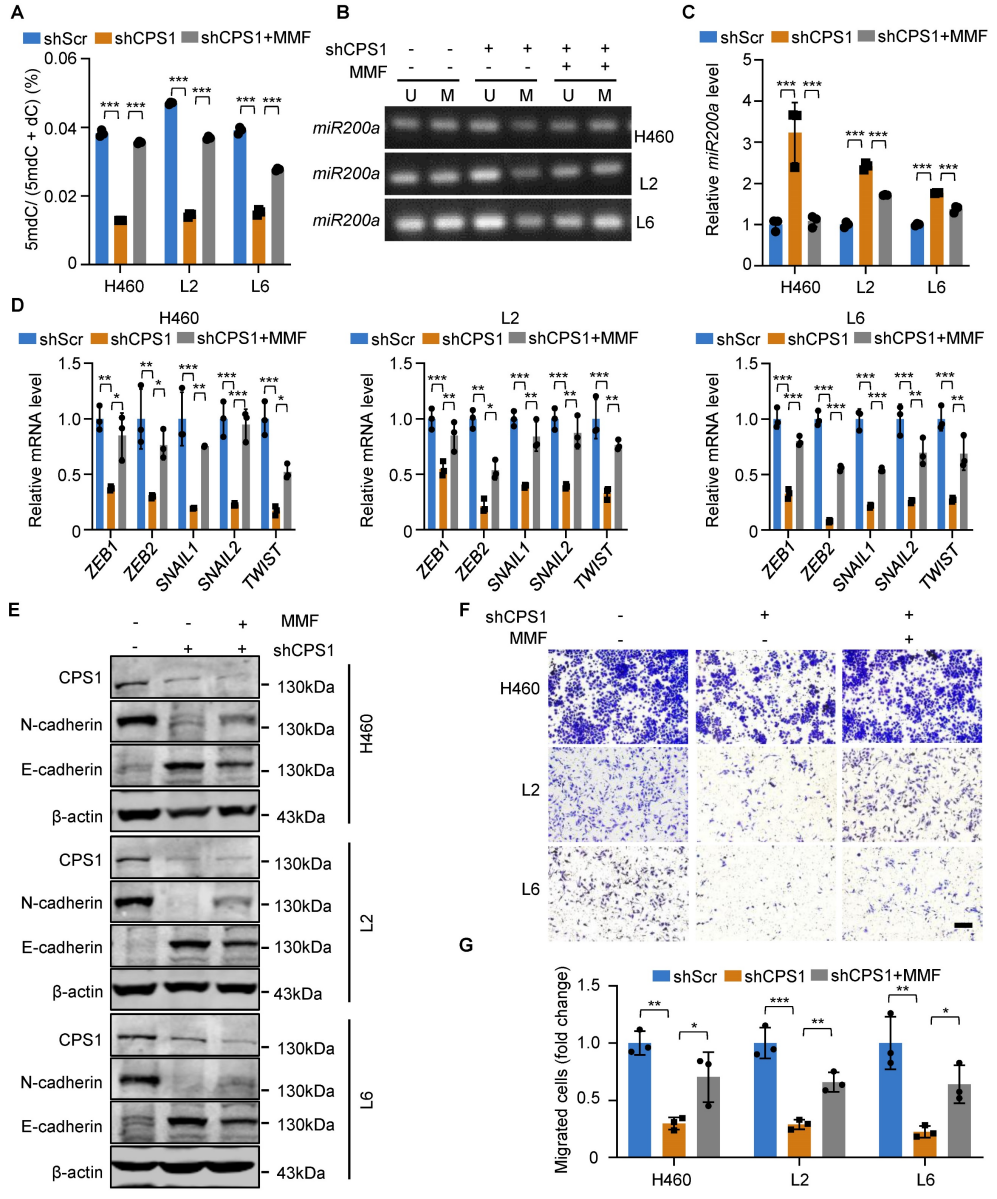
** DNA methylation and EMT in lung cancer cells are mediated by fumarate.** (**A**) Cells were treated with MMF (400 mM) or DMSO, and the contents of 5mdC and dC were measured by HPLC. n = 3. (**B**) Methylation-specific PCR of *miR200a* in metastatic cells after treatment with MMF (400 mM). (**C**) Relative mRNA expression of *miR200a* after treatment with MMF (400 mM). (**D**) Relative mRNA expression level of EMT transcription factors after treatment with MMF (400 mM) in metastatic cells. (**E**) Western blot analysis of EMT markers in metastatic cells after treatment with MMF (400 mM). (**F**) Representative images of metastatic cells after treatment with MMF (400 mM) and subjected to transwell migration assay (36 h after seeding). Scale bar: 50 μm. (**G**) Statistical analysis of the transwell assay results in (F). All the data were analyzed with unpaired two-tailed t-test. Error bars show mean ± SEM. *P < 0.05, **P < 0.01, ***P < 0.001.

Given its structural analogy to α-ketoglutarate (α-KG), we hypothesize that fumarate competitively inhibits TET2 activity, thereby promoting EMT in tumor cells via miR200a promoter methylation 32. To investigate this mechanism, we treated cells with membrane-permeable monomethyl fumarate (MMF) which is a monomethyl ester derivative of fumarate. While reducing CPS1 expression decreased 5mdC levels, treating shCPS1-expressing cells with MMF largely restored 5mdC levels (Figure 5A). Consequently, MMF treatment resulted in increased DNA methylation at the promoter region of *miR200a* (Figure 5B) and a reduced *miR200a* expression (Figure 5C). Furthermore, EMT transcription factors were all increased at the mRNA level after MMF treatment in all three metastatic cell lines (Figure 5D). Western blot analysis showed that while reducing CPS1 expression suppressed N-cadherin and restored E-cadherin expression, MMF treatment reversed the effect of CPS1 knockdown in metastatic cells (Figure 5E). This alteration of the EMT program led to a significant restoration of cell migration in MMF-treated cells, as demonstrated by the transwell assay (Figure 5F-G). These changes are all supported by alterations in fumarate level (Figure S6A). This suggests that elevated CPS1 activity drives lung cancer metastasis by accumulating fumarate to mediate the TET2 activity, miR200a DNA methylation and the EMT program.

### CPS1 promotes lung cancer metastasis *in vivo*

**Figure 6:**
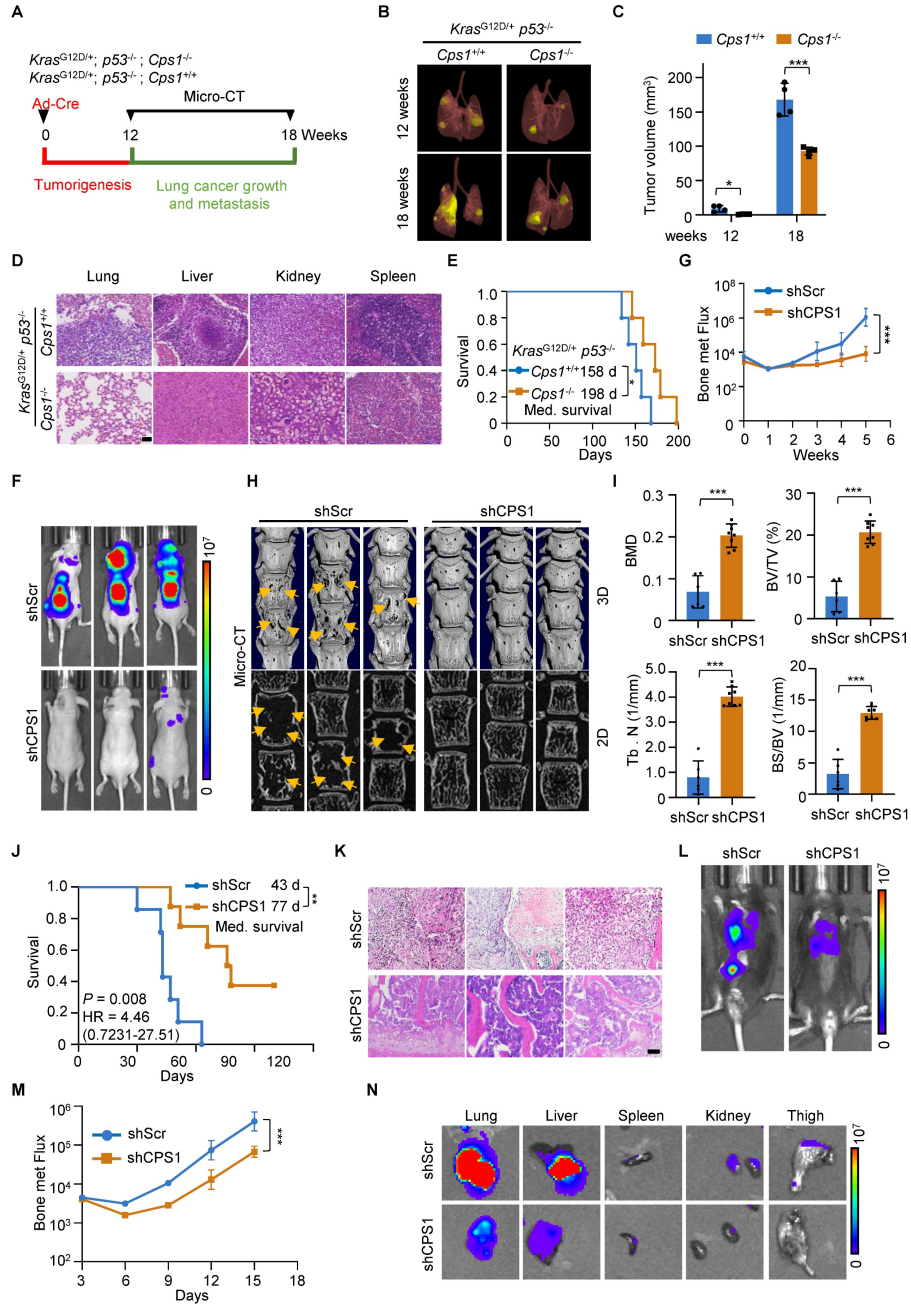
** Disruption of CPS1 activity inhibits lung cancer metastasis *in vivo*.** (**A**) Diagram of experimental design. (**B**) Representative images of tumor from KP *Cps1*^+/+^ and KP *Cps1*^-/-^ mice. n = 5 per group. (**C**) Statistics of tumor volume shown in (B). n = 4 per group. Error bars are mean ± SEM. Unpaired two-tailed t-test was used to compare groups, *P < 0.05, ***P < 0.001. (**D**) H&E staining of four organs from mice shown in (B). Scale bar: 100 μm. (**E**) Kaplan-Meier survival analysis of KP *Cps1*^*+/+*^and KP *Cps1*^*-/-*^ mice (n = 5). *P = 0.047 by log-rank test, HR = 0.2025, 95% CI of 0.04187 to 0.9797. Med., median. (**F**) Representative images of relative bioluminescence intensity (BLI) in mice injected with L6shScr (n = 6) or L6shCPS1 (n = 7), the images were captured on the 25th day post left ventricular injection. (**G**) BLI of the two groups shown in (F). BLI represents the tumor burden in the spinal bone of the mice. 2-way ANOVA was used to compare groups, ***P < 0.001. (**H**) Representative micro-CT images of the spine from the mice shown in (F) (arrows point to osteolytic areas). (**I**) Bar graph shows the quantitative micro-CT analysis of trabecular bone from the spine. BMD, bone mineral density; BV/TV, bone volume total/volume; Tb. N, trabecular number (per mm); BS/BV, bone surface/bone volume. Error bars are mean ± SEM. Unpaired two-tailed t-test was used to compare groups, ***P < 0.001. (**J**) Kaplan-Meier survival analysis of the BALB/c nude mice shown in (F). Med., median. **P = 0.008 by log-rank test. (**K**) H&E staining for bone tissues of mice shown in (F). Scale bar: 50 μm. (**L**) Representative BLI images of tumor growth and metastasis. C57BL/6 mice were injected via tail vein with either CMT167shScr (n = 5) or CMT167shCPS1 (n = 5). IVIS imaging were captured once every three days post injection, the images shown were captured on the 15th day post injection. (**M**) BLI of the two groups shown in (L). BLI represents the tumor burden of the mice. 2-way ANOVA was used to compare groups, ***P < 0.001. (**N**) *Ex vivo* fluorescence images of multiple organs from C57BL/6 mice shown in (L). Mice were sacrificed 18 days post injection and organs were dissected and BLI images were captured.

Genetically engineered mouse (GEM) model of *Kras^G12D/+^*;* p53^-/-^* (KP) is known to induce lung cancer tumorigenesis, progression and metastasis, closely mimicking the human disease 33, 34. To investigate the role of CPS1 in lung cancer progression and metastasis, we generated GEM models harboring the following alleles: (a) KP mice with wild-type *Cps1* alleles; and (b) KPC mice, which are KP mice with homozygously floxed *Cps1* alleles (Figure 6A and Figure S7A). In KPC mice, CPS1 expression was silenced in the lung of KP mouse by intranasal Adeno-Cre instillation (Figure S7B). Following Ad-Cre infection, KP mice developed substantial lung adenocarcinoma by 14 weeks (Figure 6B-C) and widespread metastatic lesions in the liver, kidney, and spleen by 18 weeks (Figure 6D), with a median survival of 158 days (Figure 6E).

In contrast, KPC mice displayed significantly reduced tumor burden, attenuated metastatic progression, and a prolonged median survival of 198 days (Figure 6E).

In the induced metastasis model, we intracardiacally injected metastatic L6 cells stably expressing either control or shCPS1 plasmids into nude mice, followed by analysis of metastatic dissemination. Bioluminescence imaging with an *In vivo* Imaging System (IVIS) demonstrated a dramatic reduction in metastasis in mice receiving shCPS1-expressing cells compared to control cells, which showed widespread dissemination and colonization of distal organs (Figure 6F and Figure S7C).

Quantitative analysis of bone metastatic flux, based on bioluminescence intensity values (BLI), indicated that the difference in relative tumor burden became evident at 3 weeks post-injection and increased significantly by 5 weeks (Figure 6G and Figure S7D). Micro-CT images of the spines revealed severe bone destruction in control mice, a phenomenon absent in mice injected with shCPS1-expressing cells (Figure 6H). Further analysis of bone damage markers, including bone mineral density (BMD); bone volume/total volume (BV/TV), trabecular number (Tb. N), and bone surface/bone volume (BS/BV), confirmed that CPS1 knockdown provided significant protection against metastatic bone damage in the spine (Figure 6I and Figure S7E). Survival analysis using the Kaplan-Meier method 35 showed that mice injected with shCPS1-expressing cells had significantly prolonged survival (average of 77 days) compared to controls (average of 43 days), with a high average hazard ratio of 12.75 (Figure 6J). Microscopic examination of H&E tissues further illustrated this, showing extensive tumor growth in the spinal bone of control mice and markedly smaller tumors in mice with shCPS1-expressing cells (Figure 6K).

To further validate CPS1's role in promoting tumor metastasis, we tested an alternative metastatic mouse model involving tail vein injection of metastatic lung cancer cells into immunocompetent C57BL/6 mouse. Mice injected with CMT167 cells expressing scrambled shRNA exhibited multi-tissue tumor growth 14 days after injection. In contrast, mice injected with CMT167 cells expressing shCPS1 displayed dramatically reduced tumor growth (Figure 6L-M). Consistently with this, bioluminescence imaging of dissected organs from C57BL/6 mice injected with shCPS1-expressing cells showed significantly reduced bioluminescent signals (Figure 6N).

Mechanistically, analysis of tumor tissues from these mice revealed an increase in mRNA expression of miR200 family members and a decrease in the mRNA expression of the EMT transcription factors following CPS1 knockdown (Figure S7F-G). This observation indicates that disruption of CPS1 inhibits lung cancer metastasis by suppressing the EMT program.

### Inhibition of CPS1 hinders lung cancer cell migration and metastasis in both induced and spontaneous metastasis mice

**Figure 7:**
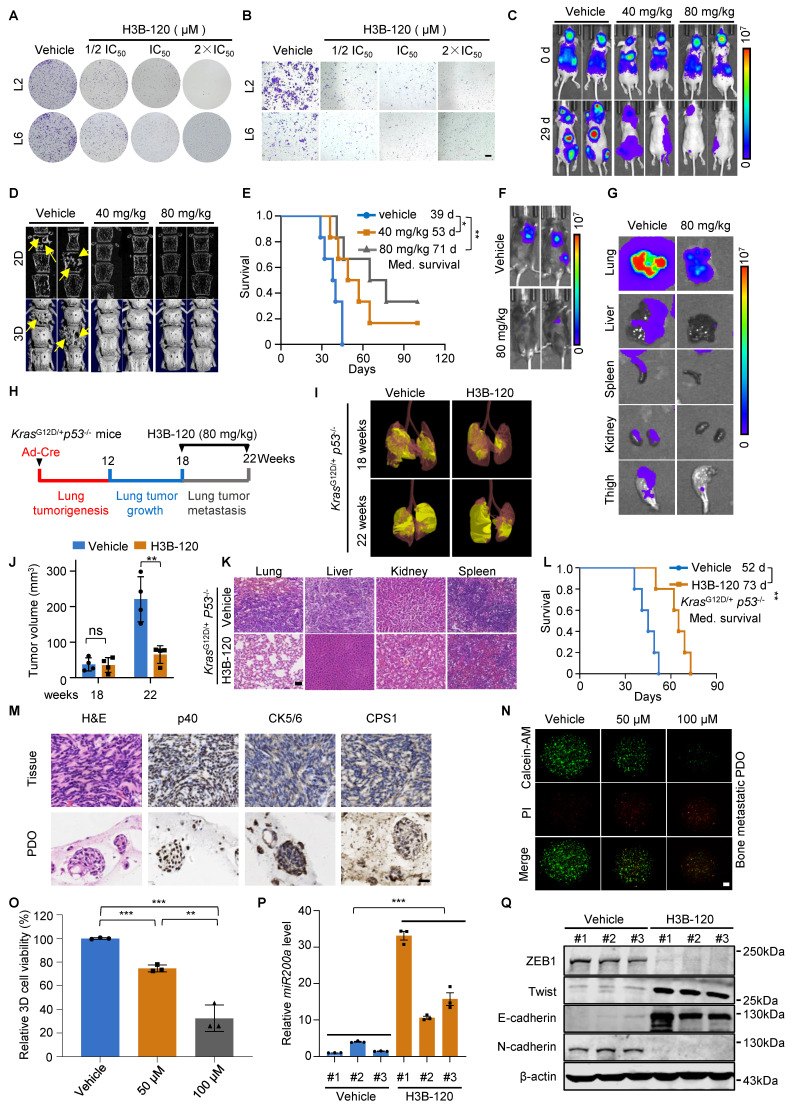
** Chemical inhibition of CPS1 suppresses lung cancer cell migration and metastasis.** (**A**) Colony formation of metastatic lung cancer cells after H3B-120 treatment. (**B**) Transwell of metastatic lung cancer cells after H3B-120 treatment. Images were captured 36 h after seeding for A and B. (**C**) Representative IVIS images illustrating tumor growth and metastasis in BALB/c nude mice after H3B-120 treatment, the images were captured on the 29th day after left ventricular injection. (**D**) Representative micro-CT images of the spine from mice shown in (C), arrows pointing to osteolytic areas. Micro-CT quantifies the relative bone volumes of the spine. (**E**) Kaplan-Meier survival analysis of BALB/c nude mice after treatment with H3B-120 (n = 6). 40 mg/kg compared to vehicle, *P = 0.0312, HR = 0.1988, 95% CI of 0.04573 to 0.8643; 80 mg/kg compared to vehicle, **P = 0.0042 by log-rank test, HR = 0.09573, 95% CI of 0.01919 to 0.4774. Med., median. (**F**) Representative BLI images of tumor growth and metastasis. C57BL/6 mice were injected with CMT167 cells via tail vein and treated with either vehicle or H3B-120 (n = 5), the images were captured on the 15th day post injection. (**G**) *Ex vivo* fluorescence images of multiple organs from C57BL/6 mice shown in (F). (**H**) Diagram of experimental design. (**I**) Representative images of tumor from KP mice after H3B-120 (80 mg/kg) treatment. (**J**) Statistics of tumor volume shown in (I). n = 4 per group. Error bars are mean ± SEM. Unpaired two-tailed t-test was used to compare groups, **P < 0.01. (**K**) H&E staining for multiple organs of mice shown in (I). Scale bar: 100 μm. (**L**) Kaplan-Meier survival analysis of KP mice after treatment with H3B-120 (n = 5). **P = 0.0064 by log-rank test, HR = 0.086, 95% CI of 0.01473 to 0.5020. Med., median. (**M**) H&E and IHC images of 3-D organoids cultured from metastatic tumor tissues of a lung cancer patient. Scale bar: 50 μm. (**N**) Immunofluorescent images of the organoids after treatment with H3B-120 for four days then stained by the Calcein/PI kit. Scale bar: 650 μm. (**O**) Relative quantification of the viability of the 3-D organoids. Error bars are mean ± SEM. *P* values were calculated using unpaired two-tailed Student's *t*-test. **P < 0.01, ***P < 0.001. (**P**) Relative mRNA expression of *miR200a* after treatment with H3B-120 (80 mg/kg). RNA was extracted from mouse tumor tissues. Error bars are mean ± SEM, **P < 0.01 value was calculated using unpaired two-tailed Student's *t*-test. (**Q**) Western blot analysis of EMT transcription factors in tumor after treatment with H3B-120 (80 mg/kg). Protein was extracted from mouse tumor tissue.

We extended the investigation to the clinical relevance and translational potential of the findings by assessing the anti-metastasis effects of the CPS1 inhibitor, H3B-120 36. *In vitro* studies determined the IC_50_ of H3B-120 to be 42 μM in L2 cells and 58 μM in L6 cells, respectively (Figure S8A). H3B-120 inhibited colony formation, indicating reduced cancer cell proliferation (Figure 7A and Figure S8B). Transwell assay further showed that H3B-120 dose-dependently inhibited migration of metastatic lung cancer cells, even concentrations below the IC_50_ (Figure 7B and Figure S8C). To translate these *in vitro* observations to an *in vivo* setting, BALB/c-nude mice with established L6 cell-induced tumors (seven days post-intracardiac injection) were treated daily with either DMSO, 40 mg/kg H3B-120, or 80 mg/kg H3B-120 via I.P. injection for three weeks. H3B-120 treatment resulted in significantly reduced tumor burden compared to DMSO-treated controls (Figure 7C and Figure S8D-F). Micro-CT scans provided further evidence, demonstrating substantial protection of spinal bones from metastatic damage by H3B-120 (Figure 7D and Figure S8G). Survival analysis using Kaplan-Meier curves indicated that H3B-120 treatment significantly prolonged survival, with average survival times of 53 days for the 40 mg/kg group and 71 days for the 80 mg/kg group, compared to 39 days for the DMSO group (Figure 7E). The inhibitory effect of H3B-120 on metastasis was further corroborated in a C57BL/6 mouse model using tail vein injection of CMT167 cells. Following daily I.P. treatment with H3B-120 for 15 days (initiated three days post-injection), mice displayed dramatically reduced tumor growth throughout the body compared to vehicle-treated controls (Figure 7F and Figure S8H). Consistently, BLI images of dissected organs showed a marked reduction in bioluminescent signal in organs from C57BL/6 mice treated with H3B-120 (Figure 7G). This compelling evidence strongly indicates that CPS1 inhibition significantly suppresses tumor metastasis.

Furthermore, we used the ICR mice to investigate the pharmacokinetics and toxicity of H3B-120. H3B-120 was well tolerated at doses up to 80 mg/kg, as it did not cause significant changes in body weight and blood biochemistry parameters (Figure S8, I-K). The half-life of H3B-120 is 0.0733 ± 0.0336 h with intravenous injection, the maximum concentration was 995 ± 128 ng/mL in concentration-time curves, and the area under the curve was 285 ± 34.7 hr*ng/mL. In comparison, the half-life of H3B-120 is 0.178 ± 0.125 h with intraperitoneal injection, the maximum concentration was 6987 ± 541 ng/mL in concentration-time curves, and area under the curve was 1355 ± 90.8 hr*ng/mL. The bioavailability of H3B-120 was 47.5%. In conclusion, H3B-120 demonstrated high systemic exposure, and intraperitoneal injection resulted in favorable absorption (Figure S8L and Table S6). Thus, it can serve as a potential lead for the treatment of metastasis of lung cancer.

In addition, we assessed the therapeutic efficacy of H3B-120 in the KP mouse model. KP mice were randomly grouped 18 weeks after intranasal administration of Ad-Cre and then treated with H3B-120 or vehicle for an additional 4 weeks (Figure 7H). H3B-120 administration significantly reduced lung tumor burden compared to the vehicle group, along with a marked suppression of metastatic progression (Figure 7I-K). Notably, H3B-120 prolonged median survival from 52 days in vehicle group to 73 days in H3B-120 group (Figure 7L). This compelling evidence shows that CPS1 inhibitor can indeed reduce tumor metastasis.

We further investigated the clinical relevance of CPS1 inhibition on patient-derived organoids (PDOs). Tumor tissues excised from bone-metastatic sites of lung cancer patients were used to establish three-dimensional organoid cultures. Histopathological analysis with H&E staining showed that the organoids retained key features of the original tumor. Furthermore, immunohistochemistry confirmed a squamous cell carcinoma phenotype, as evidenced by strong positivity for p40 and CK5/6 (Figure 7M). Importantly, positive staining for CPS1 was also observed in these PDOs (Figure 7M). We treated these P0 generation organoids with various concentrations of H3B-120 for four days. H3B-120 demonstrated a dose-dependent inhibition of metastatic organoid growth, as quantified by the cell viability marker calcein-AM (Figure 7N-O). Additional data further corroborated these findings, demonstrating that H3B-120 treatment significantly increased *miR200a* expression and suppressed EMT transcription factor expression in mouse tumor tissues (Figure 7P-Q). These results strongly suggesting that CPS1 inhibition holds promise as a therapeutic approach to suppress lung cancer metastasis.

### Targeting CPS1 enhances the effectiveness of anti-PD-1 therapy in combating lung cancer metastasis

**Figure 8:**
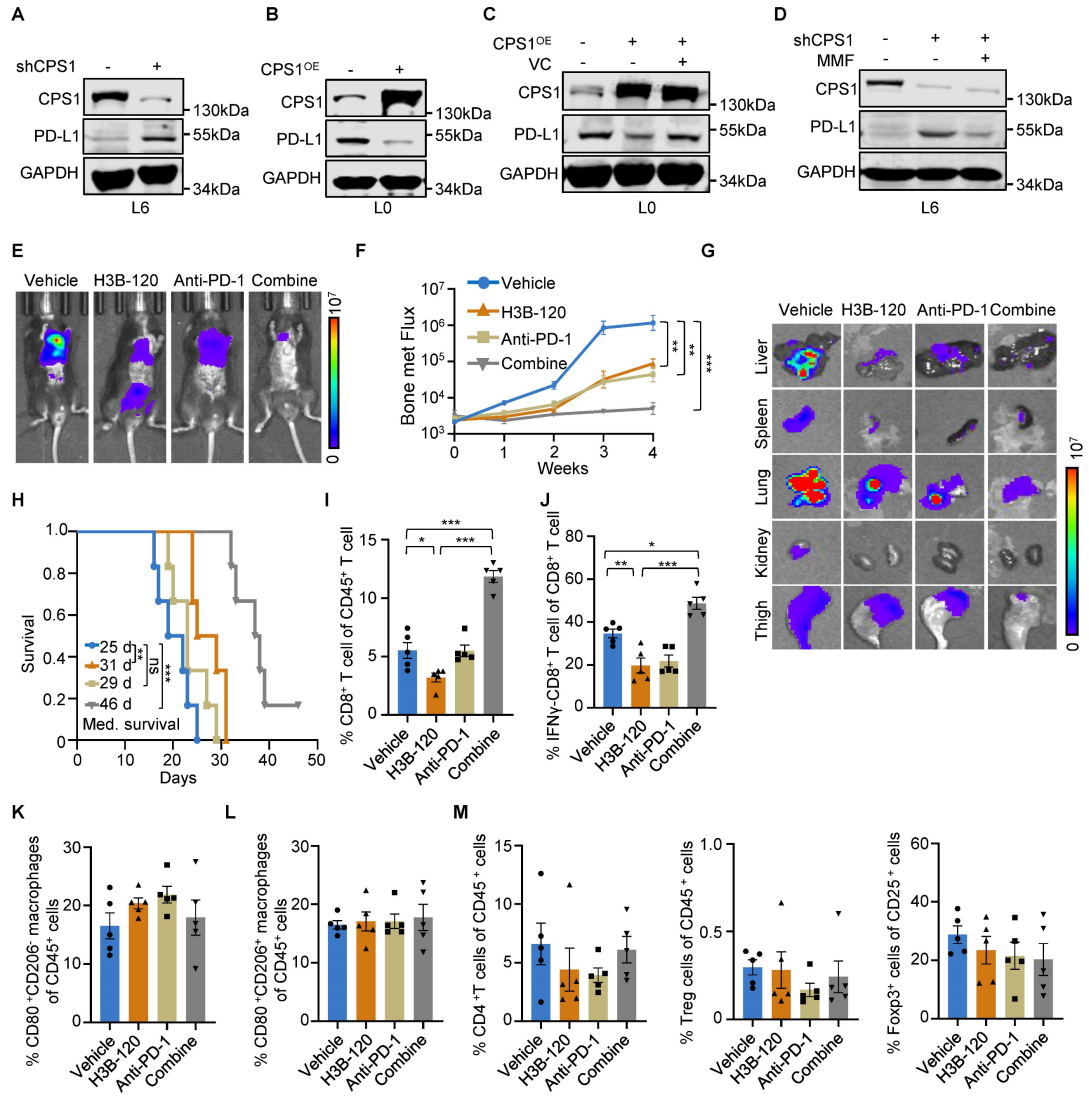
** Targeting CPS1 can sensitize lung cancer to immunotherapy.** (**A-D**) Western blot analysis. (**A**) PD-L1 expression in L6 cells after knocking down CPS1, (**B**) PD-L1 expression in L0 cells with CPS1 overexpression. (**C**) PD-L1 expression in L0 cells after over expression CPS1 and treatment with VC (250 μM)). (**D**) PD-L1 expression in L6 cells after knocking down CPS1 and treatment with MMF (400 mM)). (**E**) Representative IVIS images of indicated mice. 3 days after tail vein injection with CMT167 cells, mice were treated with H3B-120 (80 mg/kg) and/or PD-1 antibody (50 μg) for an additional 4 weeks (n = 5 per group). (**F**) BLI of the four groups shown in (E). Two-way ANOVA was used to compare groups, **P < 0.01, ***P < 0.001. (**G**) *Ex vivo* fluorescence images of multiple organs from C57BL/6 mice shown in (E). (**H**) Kaplan-Meier survival analysis of mice after treatment with H3B-120 and/or PD-1 antibody (n = 6). H3B-120 compared to vehicle **P = 0.0074 by log-rank test, HR = 3.574, 95% CI of 0.9091 to 14.05; anti-PD-1 compared to vehicle, *P* = 0.138 by log-rank test, HR = 2.084, 95% CI of 0.6222 to 6.979; combine compared to vehicle, ***P = 0.0005 by log-rank test, HR = 5.144, 95% CI of 1.138 to 23.26. (**I, J**) FACS quantitative analysis showing the treatment with H3B-120 and/or PD-1 antibody on CD8^+^ T cells and IFNγ^+^ CD8^+^ T cells within the tumor from the mice via tail vein injection. n = 5 mice per group. *P < 0.05, ***P < 0.001. *P* values were measured by one way ANOVA was used to compare groups. (**K, L**) FACS quantitative analysis shows the treatment with H3B-120 and/or PD-1 antibody on CD80^+^ macrophages and CD206^+^ CD80^+^ macrophages within the tumor from the mice via tail vein injection. n = 5 mice per group. (**M**) FACS quantitative analysis shows the treatment with H3B-120 and/or PD-1 antibody on CD4^+^ T cells, CD25^+^ T cells and Foxp3^ +^ cells within the tumor from the mice via tail vein injection. n = 5 mice per group.

Cancer immunotherapy, particularly targeting immune checkpoints, has emerged as a highly effective paradigm for treating a broad spectrum of patients, especially those who are intolerant to traditional chemotherapy. Because TET2 inhibition impairs PD-L1 transcription and confers tumor resistance to immunotherapy 29, we investigated whether augmenting TET activity through CPS1 inhibition enhances programmed death-ligand 1 (PD-L1) expression. We observed an inverse correlation, whereby reducing CPS1 expression in metastatic cells resulted in increased PD-L1 expression (Figure 8A), suggesting that CPS1 inhibition might lead to immune evasion. Conversely, overexpression of CPS1 in metastatic cells associated with a dramatic reduction in PD-L1 expression (Figure 8B). Mechanistically, this effect may involve epigenetic regulation of PD-L1, as treatment with the TET2 activator vitamin C partially rescued the reduction of PD-L1 in cells overexpressing CPS1 (Figure 8C), and treatment with the fumarate analogue MMF opposed the elevation of PD-L1 in cells with CPS1 knockdown (Figure 8D). This observation aligns with aforementioned findings demonstrating that TET2 positively regulates PD-L1 expression in some tumor types 29.

PD-L1 levels are positively correlated with immunotherapy response in several types of cancers 37-39 and high PD-L1 expression levels are associated with a better response to anti-PD-1 therapy 40. To investigate whether the CPS1 inhibition enhances immunotherapy efficacy by mediating PD-L1 upregulation, we utilized a tail vein injection model in C57BL/6 mice bearing CMT167 tumors. Mice were administered H3B-120 and/or anti-mouse PD-1 antibody. The combination treatment significantly enhanced anticancer efficacy compared with individual treatments (Figure 8E-F). Subsequent analysis of dissected organs (lung, liver, spleen, kidney, thigh) using bioluminescence intensity (BLI) images showed that the tumor area (Figure 8G) in the combination therapy group was significantly lower than in vehicle and individual treatment groups four weeks post treatment. Furthermore, Kaplan-Meier survival tests indicated that combination treatment significantly prolonged overall survival to a median of 46 days in these mice compared to the median of 25 days in control mice, and 31 days and 29 days in H3B-120-treated or anti-PD-1-treated mice, respectively (Figure 8H).

We then evaluated the antitumor immune responses within the local tumor microenvironment, using flow cytometry to analyze the changes in CD8^+^ T cell population in tumor tissues. H3B-120 treatment alone increased the CD8^+^ T cell population compared to vehicle control. This H3B-120-induced increase was more pronounced when the inhibitor was combined with an anti-PD-1 antibody (Figure 8I and Figure S9A). Although H3B-120 treatment initially reduced the activity of tumor-infiltrating cytotoxic CD8^+^ T cells, as measured by IFNγ levels, the addition of anti-PD-1 antibody restored this activity (Figure 8J and Figure S9B). Other immune cell populations, including M1 and M2 macrophages, helper T cells, regulatory T cells, all displayed insignificant changes upon H3B-120 treatment, anti-PD-1 antibody treatment, or combined treatment (Figure 8K-M and Figure S9C-F). These results demonstrate that combination treatment with anti-PD-1 therapy effectively overcomes the adverse effect of CPS1 inhibition on CD8^+^ T cells and potentiates the overall antitumor effects.

## Discussion

Previous research has highlighted the critical role of urea cycle dysregulation in tumorigenesis 41. Extending this, our study reveals for the first time that this metabolic dysregulation significantly promotes lung cancer metastasis. Overexpression of the rate-limiting enzyme CPS1 is sufficient to drive lung cancer cell migration/spreading and metastasis to distal organs. High level of CPS1 may increase urea cycle turnover rate, leading to the accumulation of its intermediate metabolite, fumarate. As an epigenetic regulator, fumarate can competitively inhibit the activity of α-KG-dependent DNA dioxygenases 17, resulting in genome-wide DNA hypermethylation. Hypermethylation of the miR200 family of tumor suppressor genes 42 leads to reduced expression of these microRNAs and, consequently, a diminished cellular ability to regulate EMT transcription factors, thus promoting the initial phase of metastasis by enhancing EMT. Critically, either reducing CPS1 expression or inhibiting its activity in metastatic cells significantly reduced metastasis of lung cancer cells in mice. Taken together, these findings not only pinpoint a novel metabolic reprogramming process driving lung cancer metastasis but also provided compelling evidence that targeting urea cycle, especially through CPS1 inhibition, could be an effective therapeutic strategy.

During metastasis, cancer cells must adapt to a host environment drastically different from their tissue of origin 43. Metabolic reprogramming is a key process enabling this adaptation 7. Shifting from catabolism to anabolism provides rapidly proliferating cancer cells with essential building blocks for macromolecule synthesis as well as helps mitigate oxidative stress 44. Consistently, dysregulation of the urea cycle has been shown to support tumorigenesis by providing excess pyrimidines and polyamines 11, 13. Under physiological conditions, CPS1 is predominantly expressed in liver cells and is generally not found in the epithelial cells of the lung. Indeed, our study detected negligible CPS1 expression in normal lung cells and low levels in para-tumor tissues or primary lung cancer cells. In contrast, CPS1 expression dramatically increased in a significant portion of metastatic lung tumors, indicating that the urea cycle underwent metabolic reprogramming and activation in these tumor cells. This research not only elucidates how the aberrant expression of a metabolic enzyme can drive the crucial initial steps of tumor metastasis, but also establishes CPS1-mediated urea cycle reprogramming as a druggable metabolic vulnerability for combating metastatic disease.

Numerous tumorigenesis drivers have been identified, primarily somatic mutations in oncogenes and tumor suppressor genes 45. Addressing the driving forces behind tumor metastasis have been an active area of investigation in cancer biology, utilizing various experimental techniques like single-cell RNA-sequencing, cellular lineage tracing, patient-derived xenografts, and computational approaches 46. However, large-scale genomic analysis of metastatic tumor tissues has revealed a mutational landscape largely similar to their primary tumors, without identifying mutations exclusively driving metastasis 47-49. Despite this, recent genomic analyses of NSCLC have identified increased mutation burden in brain metastases compared to primary tumors, particularly in genes such as *CDKN2*, *TP53*, *KRAS*, and *EGFR* 50, a finding consistent with pan-cancer whole-genome analysis 47. Given that mutations in *KRAS/LKB1* or *TP53* are known upstream drivers of CPS1 overexpression 11, 13, our finding that CPS1 overexpression promotes lung cancer metastasis can be considered as a functional consequence of these driver gene mutations. Our retrospective analysis of patient samples observed that metastatic patients harboring *KRAS* mutations tended to exhibit higher CPS1 protein levels compared to those with wild-type *KRAS*, aligning with the known role of *KRAS/LKB1* mutations in driving CPS1 expression. Therefore, we assume that patients with KRAS-mutant metastatic lung cancer exhibiting high CPS1 protein expression are most likely to benefit from CPS1-targeted therapies. However, future studies with larger patient cohorts are necessary to validate which patient populations would benefit most from this therapeutic strategy.

Recent studies have demonstrated that dysregulation of the urea cycle promotes T cell exhaustion, contributing to immunotherapy failure 51. This finding suggests that targeting urea cycle may represent a promising strategy to enhance immunotherapy efficacy. In this study, CPS1 is highly expressed in metastatic lung cancer cells and elevated CPS1 activity accumulates urea cycle-derived fumarate to inhibit TET2 activity, which regulates miR200a-EMT axis in tumor metastasis and reduces PD-L1 in tumor immunity. Previous studies have shown that TET2 mediates the IFN-γ/JAK/STAT signaling axis to regulate PD-L1 expression, lymphocyte infiltration dynamics, and anti-tumor immunity 29. This observation aligns with our findings, indicating that CPS1 depletion upregulates PD-L1 through fumarate-mediated modulation of TET2 activity, thereby potentially promoting tumor immune evasion. Importantly, immune profiling of tumors following CPS1 inhibition (H3B-120) revealed no significant alterations in the infiltration of macrophages or Tregs, but specifically suppressed the abundance and activity of CD8⁺ T cells. Combining CPS1 inhibition and PD-1 blockade effectively suppressed lung cancer metastasis and significantly prolonged the survival of mice, by directly targeting tumor cells via the metabolic pathway while concurrently reinvigorating the anti-tumor activity of CD8⁺ T cells. A recent study revealed that CD8⁺T cells can acquire urea cycle function through the expression of urea cycle-related enzymes 52, further confirming the close relationship between urea cycle and tumor immunity. Together, our study also highlights that urea cycle acts as a metabolic checkpoint for controlling immune responses in the tumor microenvironment.

Our findings demonstrate that reducing CPS1 expression, either through genetic manipulation or enzymatic inhibition, significantly reduces lung cancer metastasis in mice, establishing a robust foundation for future translational research. Notably, applying the CPS1 inhibitor to metastatic tumor cells in circulation, prior to colonization, effectively protected bones from metastatic damage. Furthermore, the inhibitor suppressed the growth of organoids derived from metastatic bone tumor tissue of a lung cancer patient. While this inhibitory effect in organoids likely targeted tumor growth rather than metastasis directly, consistent with previous studies 12, it suggests the translational potential of CPS1 inhibitors for lung cancer patients presenting with early signs of metastasis. However, a limitation is the high half maximal inhibitory concentration of the current inhibitor, posing a substantial obstacle for clinical application. Further efforts should prioritize enhancing its efficacy through computer-aided drug design to modify H3B-120's molecular structure. Alternatively, a virtual screening of existing clinically approved or trialed drugs for potent CPS1 inhibitors could expedite translation. Another limitation is that CPS1 overexpression occurs only in a subset of metastatic lung cancer patients. Furthermore, the therapeutic targeting of CPS1, like other targeted therapies, may face the challenge of drug resistance. Metabolic plasticity is a key resistance mechanism, as evidenced by reports that CPS1 loss can induce sorafenib resistance and activate compensatory Fatty Acid β-Oxidation (FAO) to sustain tumor growth 14. Therefore, rational combination strategies, such as pairing CPS1 inhibitors with FAO pathway blockers or immune checkpoint inhibitors, is a promising direction to overcome potential resistance and achieve durable therapeutic efficacy. Nevertheless, identifying overexpressed CPS1 and activated urea cycle in lung cancer patients, ideally at localized stage, offers a promising opportunity for personalized treatment strategies to improve patient outcomes.

## Conclusion

In summary, this study identified the urea cycle enzyme CPS1 as a pivotal promoter of lung cancer metastasis through a defined metabolic-epigenetic pathway. We found that CPS1 upregulation leads to the accumulation of fumarate, which acts as an oncometabolite to inhibit TET2 activity. This inhibition results in hypermethylation and silencing of the *miR200a* gene, thereby unleashing EMT transcription factors and enhancing the invasive capacity of cancer cells. Importantly, either genetic knockdown or pharmacological inhibition of CPS1 robustly attenuated metastatic progression across multiple *in vivo* models. Furthermore, combining a CPS1 inhibitor with anti-PD-1 therapy yielded a synergistic antitumor effect, overcoming immune evasion. Collectively, our work establishes CPS1 as a critical metabolic driver of metastasis and a viable therapeutic target for lung cancer metastasis.

## Supplementary Material

Supplementary figures.

Supplementary tables.

## Abbreviations

ASL: Argininosuccinate lyase
ASS1: Argininosuccinate synthetase 1
AST: Aspartate Aminotransferase
ALT: Alanine Aminotransferase
CPTAC: Clinical Proteomic Tumor Analysis Consortium
EMT: Epithelial-mesenchymal transition
EDTA: ethylenediaminetetraacetic acid
FAO: Fatty Acid β-Oxidation
FH: fumarate hydratase
FBS: fetal bovine serum
PDO: Patient-derived organoid
IC50: half maximal inhibitory concentration
HLRCC: Hereditary leiomyomatosis and renal cell cancer
MMF: Monomethyl fumarate
TCGA: The Cancer Genome Atlas
LUAD: Lung Adenocarcinoma
LUSC: Lung squamous cell carcinoma
TCA: Tricarboxylic Acid Cycle
TET: Ten-Eleven Translocation
PFA: paraformaldehyde
5hmC: 5-hydroxymethylcytosine
5mC: 5-methlycytosine
5mdC: 5-methyl-deoxycytidine
OGDH: α-ketoglutarate dehydrogenase
α-KG: α-ketoglutaric acid
