# Supplementary material for: Targeting CPS1 attenuates lung cancer metastasis by regulating EMT through an epigenetic mechanism
Source: Theranostics. 2026 Jan 1;16(4):1740–61. doi: 10.7150/thno.123679 (PMC12680530; doi:10.7150/thno.123679)
Supplement: Supplementary file 1 — Supplementary figures. [file thnov16p1740s1.pdf]

This supporting information contains

1. Figure S1 supporting information. CPS1 is overexpressed in metastatic lung cancer and correlates with poor prognosis. Related to Figure 1.

2. Figure S2 supporting information. Reducing CPS1 expression inhibits cancer cell migration and EMT.

3. Figure S3 supporting information. CPS1 Promotes EMT by Silencing miR-200a. Related to Figure 3. Related to Figure 3.

4. Figure S4 supporting information. CPS1 promotes EMT by TET2-induced miR200a methylation. Related to Figure 3.

5. Figure S5 supporting information. The level of fumarate is regulated by CPS1 through urea cycle. Related to Figure 4.

6. Figure S6 supporting information. DNA methylation and EMT in lung cancer cells are mediated by fumarate. Related to Figure 5.

7. Figure S7 supporting information. Disruption of CPS1 activity inhibits lung cancer metastasis *in vivo*. Related to Figure 6.

8. Figure S8 supporting information. Chemical inhibition of CPS1 suppresses lung cancer cell migration and metastasis. Related to Figure 7.

9. Figure S9 supporting information. Targeting CPS1 can sensitize lung cancer to immunotherapy. Related to Figure 8.

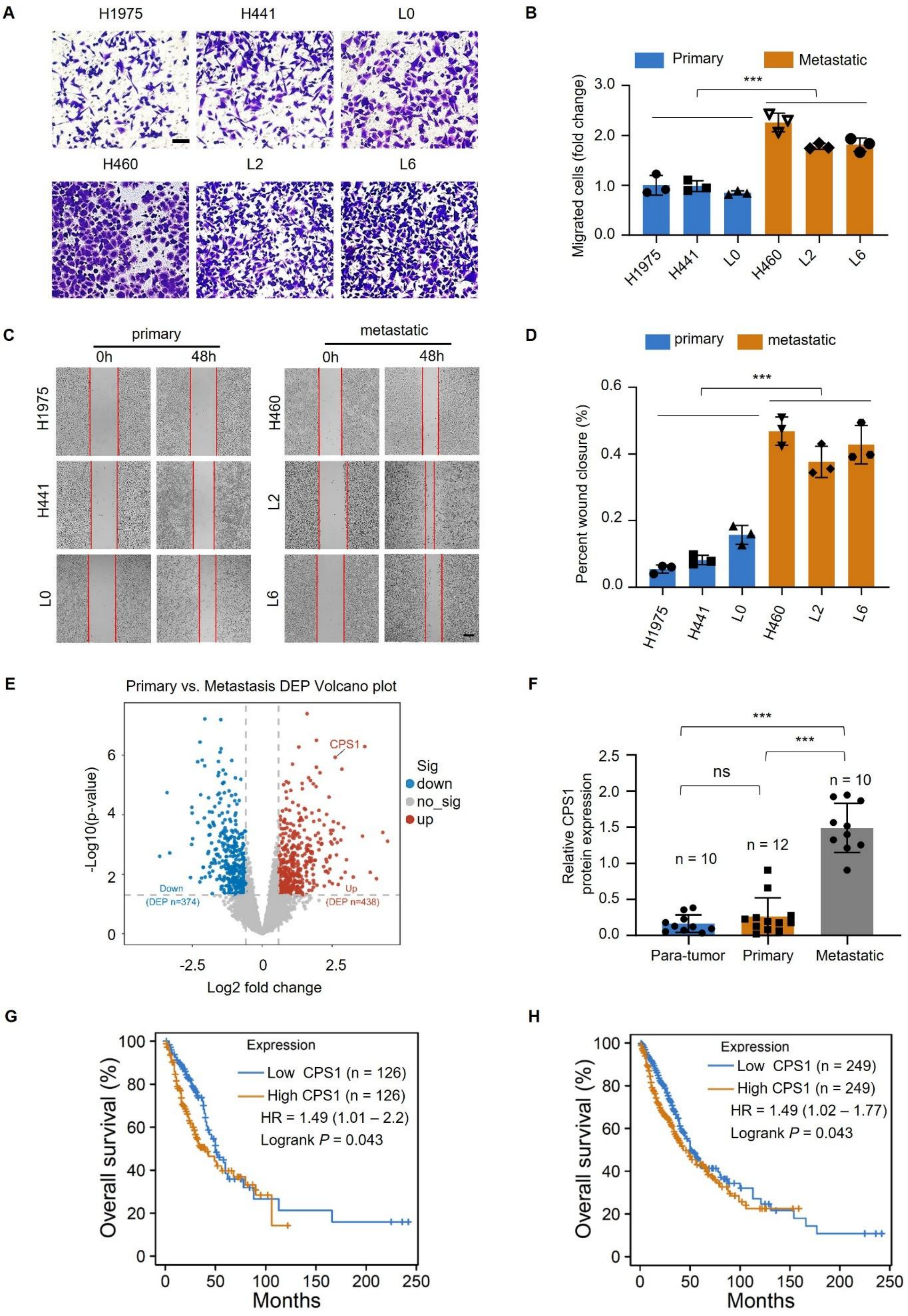

A
H1975
H441
L0
H460
L2
L6
B
Primary
Metastatic
Migrated cells (fold change)
***
C
primary
metastatic
0h
48h
D
Percent wound closure (%)
E
Primary vs. Metastasis DEP Volcano plot
CPS1
Sig
down
no_sig
up
-Log10(p-value)
Log2 fold change
Down (DEP n=374)
Up (DEP n=438)
F
Relative CPS1 protein expression
ns
n = 10
n = 12
n = 10
Para-tumor
Primary
Metastatic
G
Expression
Low CPS1 (n = 126)
High CPS1 (n = 126)
HR = 1.49 (1.01 – 2.2)
Logrank P = 0.043
Overall survival (%)
Months
H
Expression
Low CPS1 (n = 249)
High CPS1 (n = 249)
HR = 1.49 (1.02 – 1.77)
Logrank P = 0.043
Overall survival (%)
Months

Figure S1. CPS1 is overexpressed in metastatic lung cancer and correlates with poor prognosis. Related to Figure 1. (**A**) Representative images of transwell assay comparing primary and metastatic cells (36 h after seeding). Scale bar: 200 μm. (**B**) Statistical analysis of the transwell assay results in (A). ****P* value was obtained using unpaired two-tailed t-test. (**C**) Representative images of wound healing assay comparing primary and metastatic cells (48 h after seeding). (**D**) Statistical analysis of the wound healing results in (C). ****P* value was obtained using unpaired two-tailed t-test. (**E**) Volcano plot of significantly changed proteins in metastatic comparing to primary lung cancer cells. Related to Figure 1A. (**F**) Statistical analysis of the western blot assay results in Figure 1F. Unpaired two-tailed t-test was used to compare the groups. **$P < 0.01$. (**G**) and (**H**) Kaplan-Meier survival analysis comparing patient survival between CPS1 high expression versus low expression using 50% as the expression cutoff. For survival analysis log-rank test was used, for HR Cox proportional hazard model was used. Data from lung cancer patients was downloaded from TCGA.

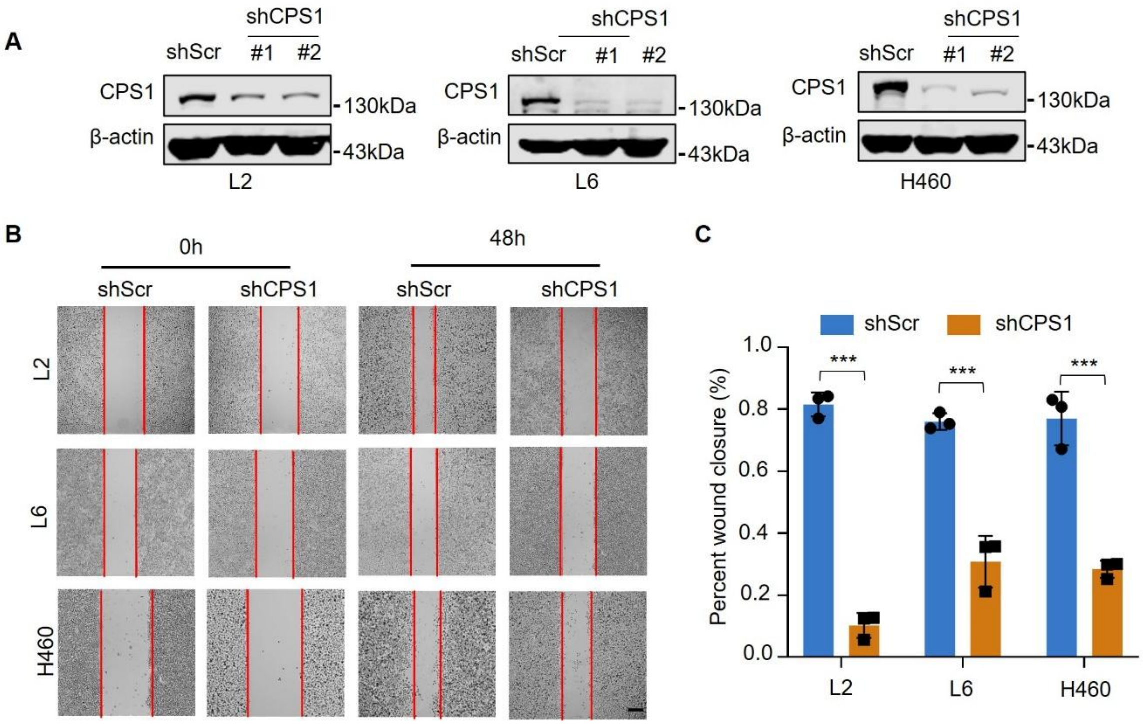

Figure S2. Reducing CPS1 expression inhibits cancer cell migration and EMT. Related to Figure 2. (A) Western blot analysis of CPS1 expression after knocking down CPS1. (**B** and **C**) Wound healing assay. The wound areas after cell migration were analyzed using image J. Error bars are mean ± SEM. *P* values were obtained using unpaired two-tailed t-test. ****P* < 0.001.

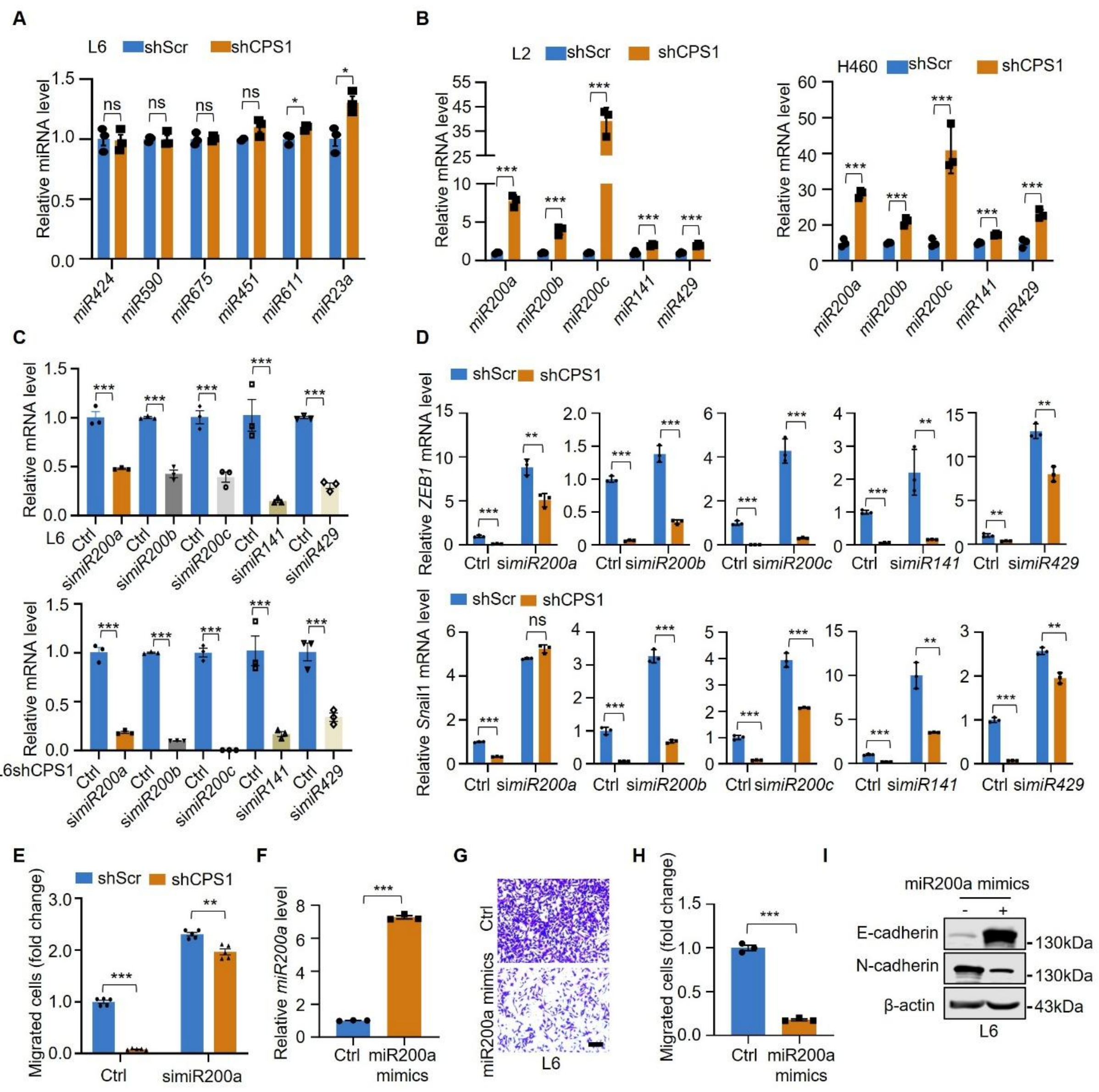

Figure S3. CPS1 Promotes EMT by Silencing miR-200a. Related to Figure 3. (A) Relative expression levels of *miR424, miR590, miR675, miR611, and miR23a* in L6 and L6shCPS1 cells. (**B**) Relative expression levels of *miR200* family members in L2 and H460 cells after CPS1 knockdown. (**C**) QPCR analysis showed that *miR200* family members were silenced in L6 and L6shCPS1 cells. (**D**) QPCR analysis showed the expression levels of *ZEB1* and *SNAIL1* after silencing miR200 family members in L6 and L6shCPS1 cells. (**E**) Statistical analysis of the transwell assay results in Figure 3F. (**F**) Relative expression levels of *miR200a* in L6 and L6 overexpression of miR200a. (**G**) Representative images of transwell assay showing cell migration after overexpression of miR200a in L6 cells. Images were taken 36 h after seeding. Scale bar: 200 μm. (**H**) Statistical analysis of the transwell assay results in (G). (**I**) Western blot analysis of EMT markers in L6 cells with miR200a overexpression. All data were analyzed with unpaired two-tailed t-test. Error bars show mean ± SEM. *$P < 0.05$,

$^{**}P < 0.01$, $^{***}P < 0.001$.

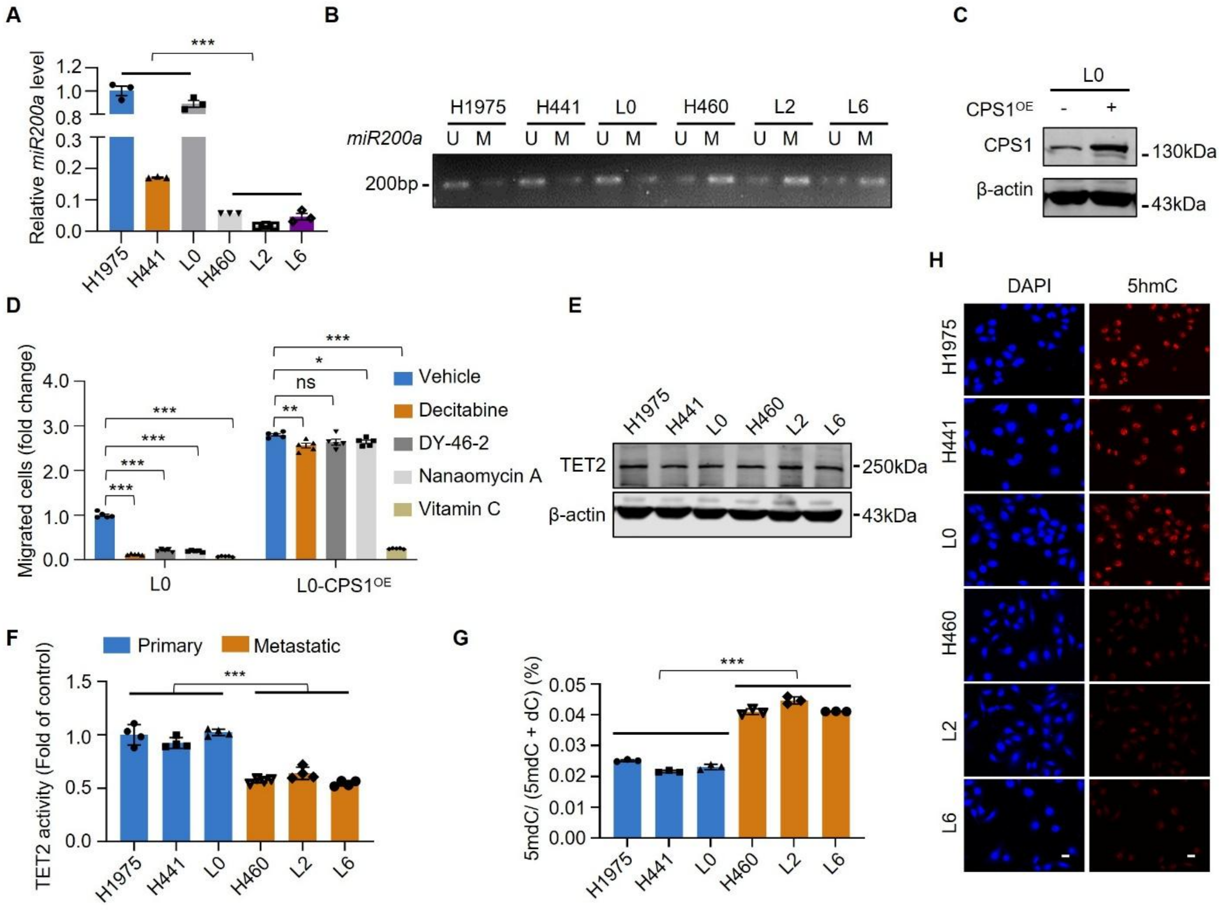

Figure S4. CPS1 promotes EMT by TET2-induced *miR200a* methylation. Related to Figure 3. (**A**) Relative expression levels of miR200a in primary and metastatic cancer cell lines. (**B**) Methylation-specific PCR of miR200 family members in primary and metastatic cancer cells. U: un-methylated; M: methylated CpG island. qPCR results were obtained from at least 3 independent cell cultures. (**C**) Western blot analysis showed that CPS1 was overexpressed in L0 cells. (**D**) Statistical analysis of the transwell assay results in Figure 3J. (**E**) Western blot analysis of TET2 expression in primary and metastatic cancer cell lines. (**F**) Enzymatic activity of TET2 in primary and metastatic cancer cell lines. (**G**) Quantification of 5-methyl-deoxycytosine (5mdC) and deoxycytosine (dC) in primary and metastatic lung cancer cells, n = 3. (**H**) Immunofluorescent imaging of 5hmC (red) and DAPI staining (blue) after knocking down CPS1 in multiple lung cancer cell lines.

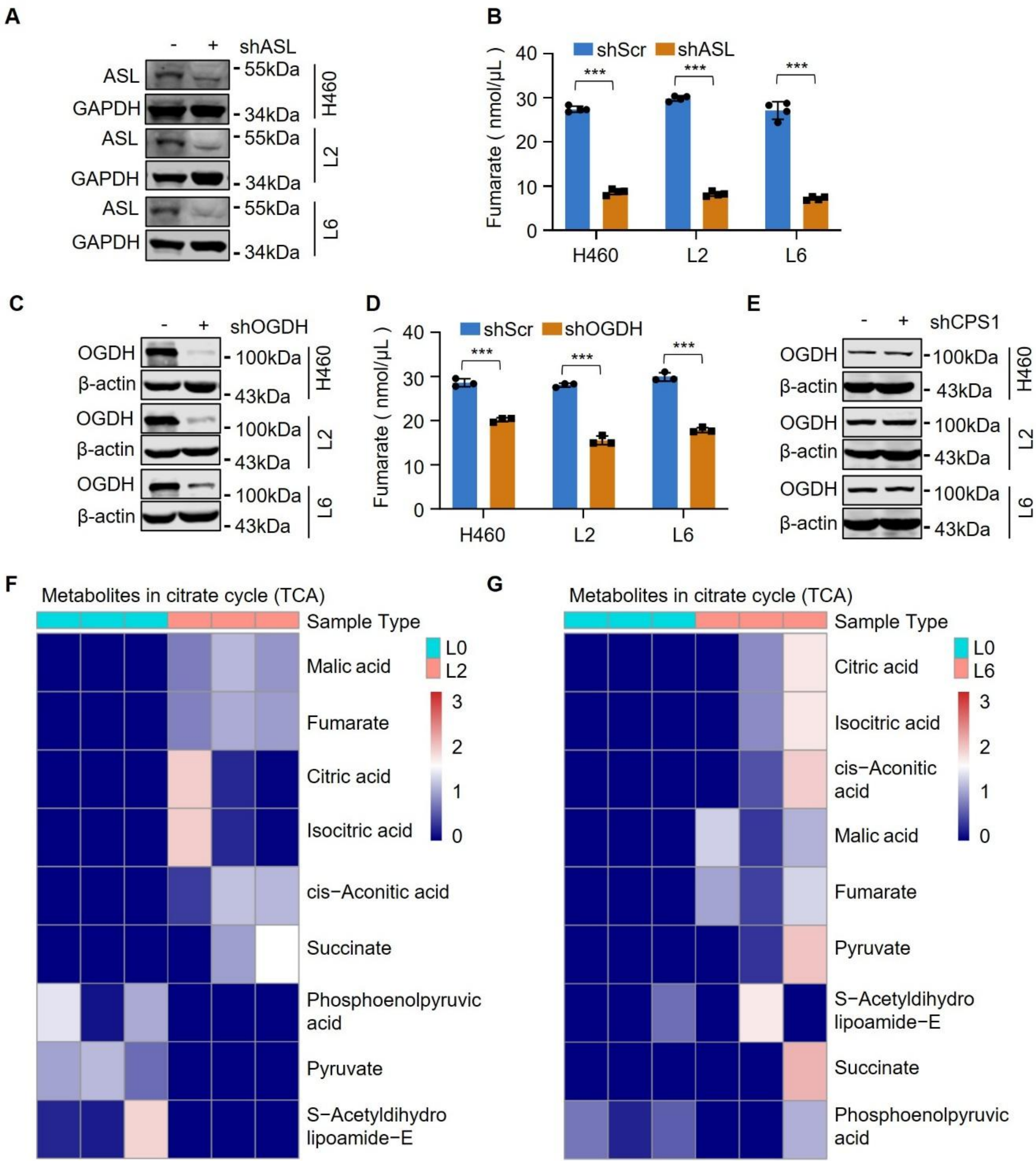

Figure S5. The level of fumarate is regulated by CPS1 through urea cycle. Related to Figure 4. (**A**) Western blot analysis showing ASL expression after knocking down in metastatic cancer cells. (**B**) The level of fumarate after knocking down ASL in metastatic lung cancer cells. n = 3. *P* values were obtained using unpaired Student's t-test. ****P* < 0.001. (**C**) Western blot analysis showing OGDH expression after knocking down in metastatic cancer cells. (**D**) The level of fumarate after knocking down OGDH in metastatic lung cancer cells. n = 3. *P* values were obtained using unpaired Student's t-test. ****P* < 0.001. (**E**) Western blot analysis of OGDH after knocking down CPS1 in metastatic lung cancer cell lines. (**F**) Heatmap illustrating the ratios of representative

metabolites enriched in TCA cycle KEGG pathway (L0 vs L2). n = 3 for per group. (**G**) Heatmap illustrating the ratios of representative metabolites enriched in TCA cycle KEGG pathway (L0 vs L6). n = 3 for per group.

A

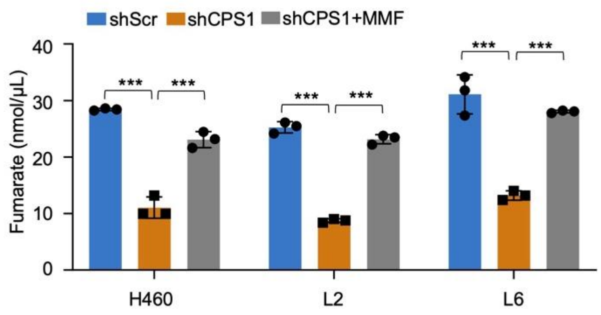

Figure S6. DNA methylation and EMT in lung cancer cells are mediated by fumarate. Related to Figure 5. (**A**) The level of fumarate in metastatic lung cancer cells after knocking down CPS1 and treatment with MMF. P values were obtained using unpaired Student's t-test. ***$P < 0.001$.

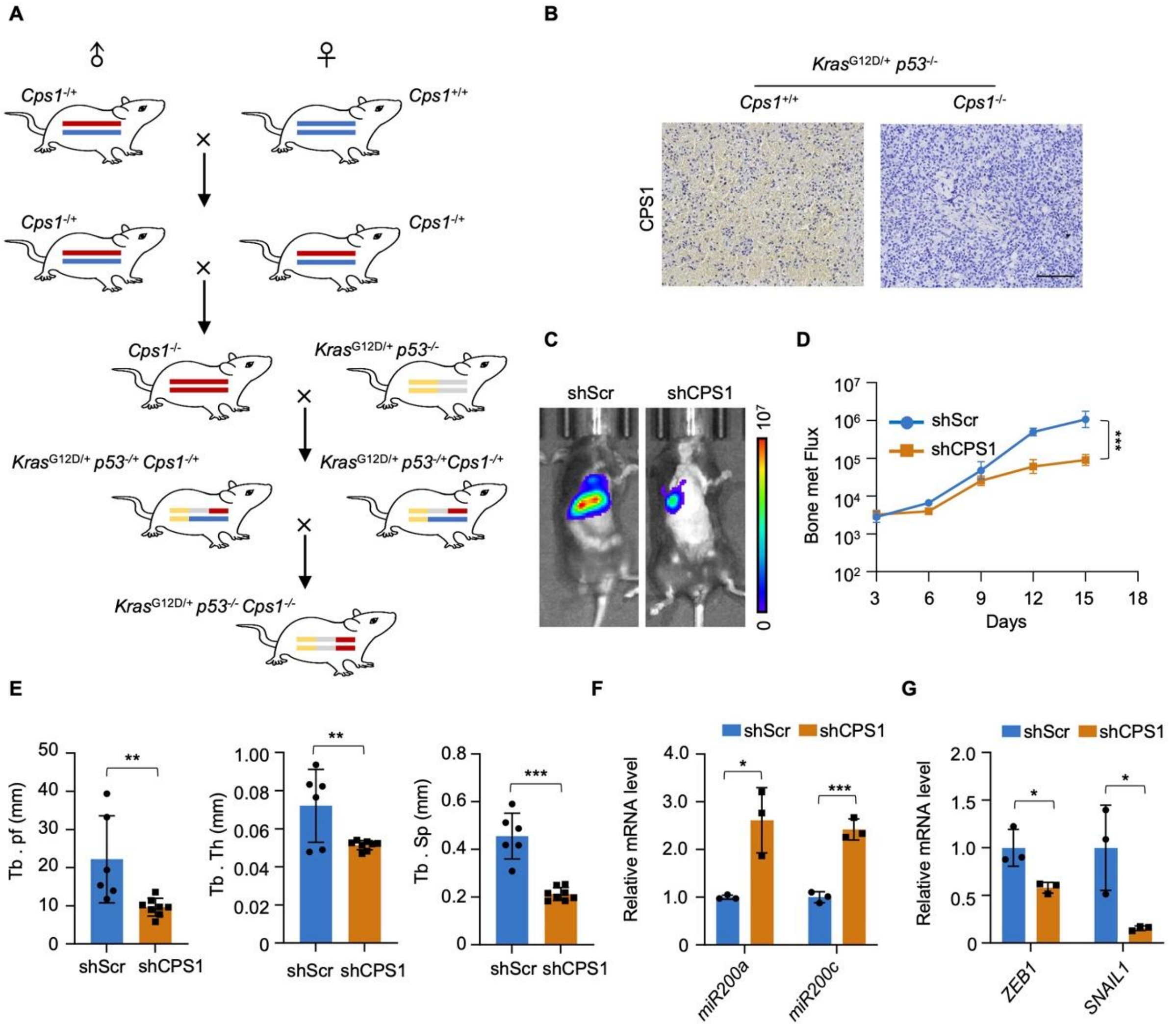

Figure S7. Disruption of CPS1 activity inhibits lung cancer metastasis *in vivo*. Related to Figure 6. (**A**) Schematic illustration of LoxP-stop-loxP *Kras*$^{G12D/+}$; *Trp53*$^{-/-}$; *Cps1*$^{-/-}$ (KP *Cps1*$^{-/-}$) mouse model. (**B**) Representative CPS1 staining results of lung tumors in KP *Cps1*$^{+/+}$ and KP *Cps1*$^{-/-}$ mice. Scale bar: 100 μm. (**C**) Representative images of relative bioluminescence intensity (BLI) in female mice injected with CMT167shScr or CMT167shCPS1 (n = 5). (**D**) BLI of the two groups shown in (C). BLI represents the tumor burden of the mice. 2-way ANOVA was used to compare groups, ****P* < 0.001. (**E**) Bar graph of quantitative micro-CT analysis of trabecular bone from the spine. Tb.pf, trabecular pattern factor; Tb.Th, trabecular thickness; Tb.Sp, trabecular separation. *P* values were obtained using unpaired Student's t-test. ***P* < 0.01, ****P* < 0.001. (**F**) QPCR analysis of *miR200* family members. n = 3. RNA was extracted from mouse tumor tissues shown in figure6 (L). (**G**) QPCR analysis of mRNAs of EMT transcription factors. n = 3. RNA was extracted from mouse tumor tissue shown in figure6 (L). *P* values were obtained using unpaired Student's t-test. **P* < 0.05, ****P* < 0.001.

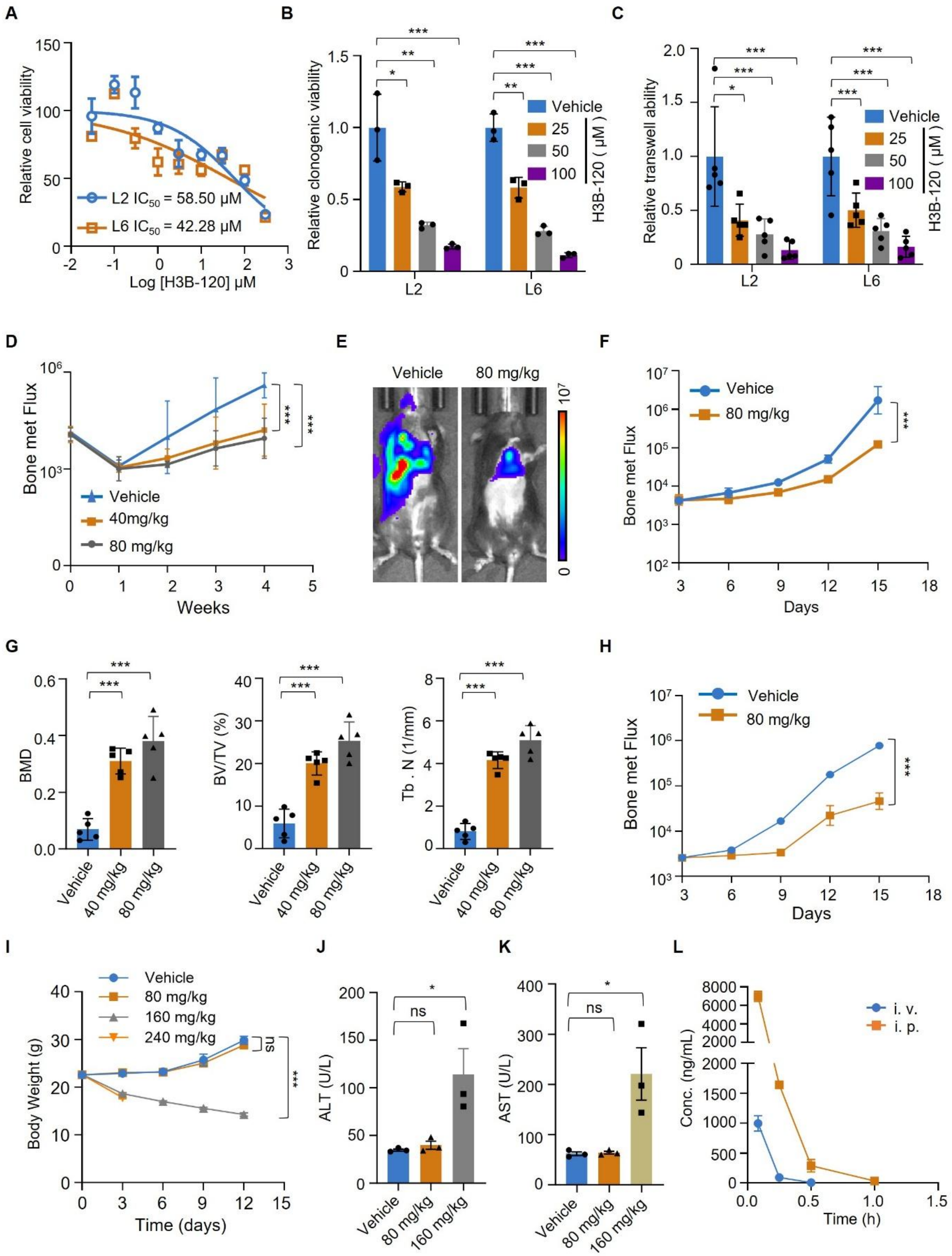

Figure S8. Chemical inhibition of CPS1 suppresses lung cancer cell migration and metastasis. Related to

Figure. 7. (**A**) IC_{50} of H3B-120 in L2 and L6 cells. (**B**) Statistical analysis of colony formation assay results after H3B-120 treatment., *P* values were obtained using unpaired Student's t-test. *$P < 0.05$, **$P < 0.01$, ***$P < 0.001$. n = 3. (**C**) Statistical analysis of transwell assay results after H3B-120 treatment. *P* values were obtained using unpaired Student's t-test. *$P < 0.05$, ***$P < 0.001$. n = 3. (**D**) Bar graph showing the relative bioluminescence intensity (BLI) of tumors after intracardiac injection shown in figure7. (C). The data was analyzed using 2-way ANOVA, ***$P < 0.001$.n = 8. (**E**) Representative BLI images of tumor growth and metastasis. C57 female mice were injected with CMT167 cells via tail vein and treated with either vehicle or H3B-120 (n = 5), the images were captured on the 15th day post injection. (**F**) BLI of the four groups shown in (E). 2-way ANOVA was used to compare groups, ***$P < 0.001$. (**G**) Micro-CT analysis of BMD shown in figure7 (D), BV/TV, Tb. N. *P* values were obtained using unpaired Student's t-test, **$P < 0.01$, ***$P < 0.001$. Error bars represent mean ± SEM (n = 5). (**H**) Bar graph showing the BLI via tail vein injection shown in figure7 (F). BLI of the mice were collected every three days. 2-way ANOVA was obtained using unpaired Student's t-test, ***$P < 0.001$. Error bars represent mean ± SEM. (**I**) The mouse body weight in the ICR mice (n = 5). (**J, K**) Effects of H3B-120 on clinical chemistry analysis in mice (n =3). Serum samples were collected from 6-week-old ICR mice. Liver function indexes include ALT and AST. The value is shown as mean ± SEM. *$P < 0.05$, **$P < 0.01$, and ***$P < 0.001$. (**L**) The logarithmic semi-logarithmic curve of blood drug concentration-time for H3B-120.

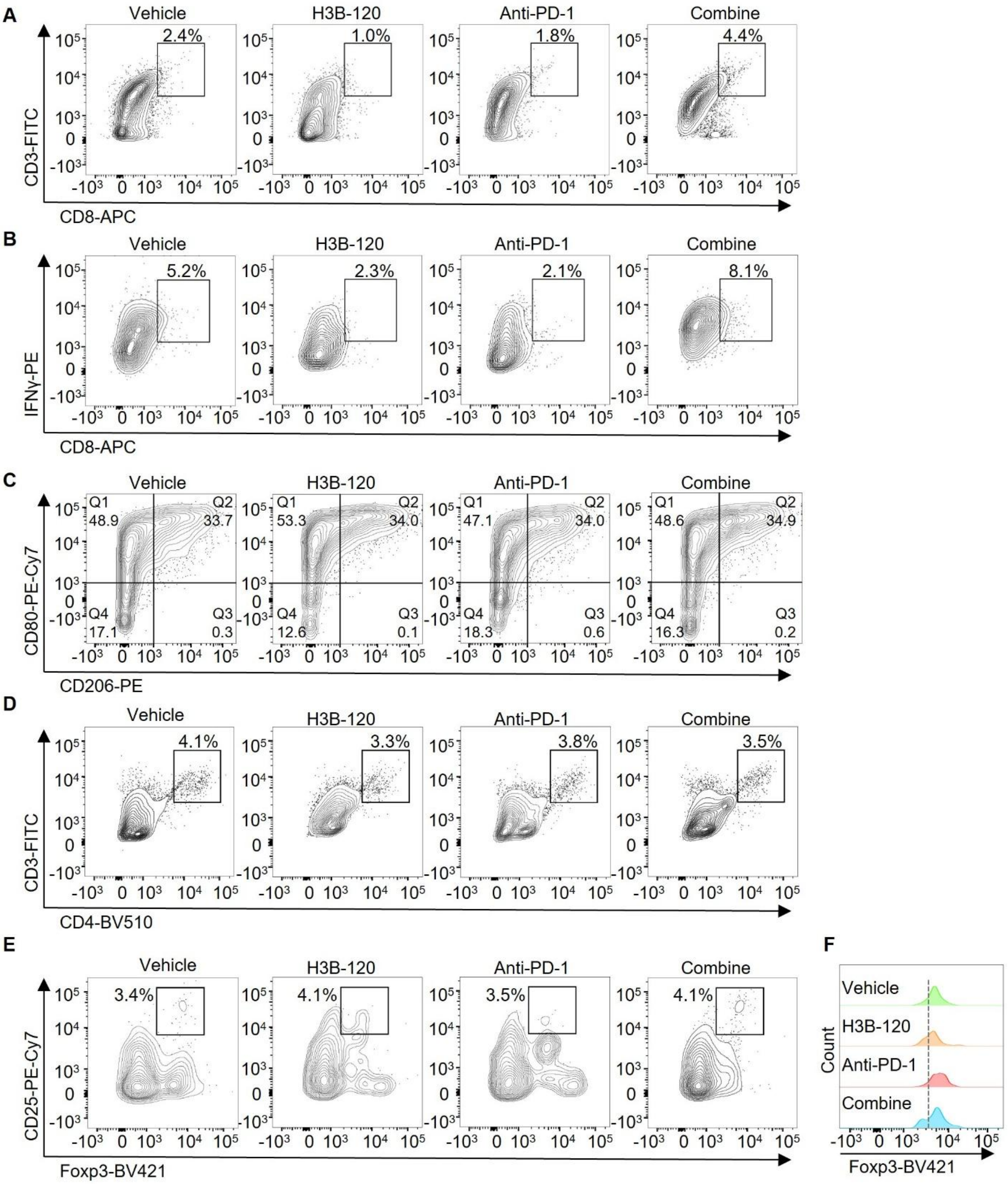

Figure S9. Targeting CPS1 sensitizes lung cancer to immunotherapy. Related to Figure 8. (**A and B**) Representative FACS plots of CD8$^+$ in CD45$^+$ cells and IFNγ$^+$ in CD8$^+$ T cells within CMT167 tumors from the mice following tail vein injection, with or without H3B-120 and anti-PD-1 treatment. (**C**) Representative FACS plots of CD80$^+$ CD206$^+$ macrophages within CMT167 tumors from the mice following tail vein injection, with or without H3B-120 and anti-PD-1 treatment. (**D and E**) Representative FACS plots of CD4$^+$ T cells, CD25$^+$ T

cells and Foxp3^{+} cells within CMT167 tumors from the mice following tail vein injection, with or without H3B-120 and anti-PD-1 treatment. (**F**) Representative histogram plots of Foxp3 expression within macrophages within CMT167 tumors from the mice following tail vein injection, with or without H3B-120 and anti-PD-1 treatment.
